# Comprehensive analysis of FRAS1/FREM family as potential biomarkers and therapeutic targets in renal clear cell carcinoma

**DOI:** 10.3389/fphar.2022.972934

**Published:** 2022-09-29

**Authors:** Ganggang Wang, Zheng Wang, Haiquan Lu, Zhiqun Zhao, Liqiang Guo, Feng Kong, Aizhen Wang, Shengtian Zhao

**Affiliations:** ^1^ Department of Urology, Shandong Provincial Hospital, Shandong University, Jinan, Shandong, China; ^2^ Department of Urology, Maternal and Child Health Care Hospital of Shandong Province, Shandong University, Jinan, Shandong, China; ^3^ Advanced Medical Research Institute and Key Laboratory for Experimental Teratology of the Ministry of Education, Cheeloo College of Medicine, Shandong University, Jinan, Shandong, China; ^4^ Department of Urology, Qilu Hospital, Shandong University, Jinan, Shandong, China; ^5^ Binzhou Medical University, Binzhou, Shandong, China

**Keywords:** renal clear cell carcinoma, prognosis, biomarker, therapeutic target, tumor immune infiltrating, DNA methylation

## Abstract

**Background:** FRAS1 (Fraser syndrome protein 1), together with FREM1 (the Fras1-related extracellular matrix proteins 1) and FREM2, belonging to the FRAS1/FREM extracellular matrix protein family, are considered to play essential roles in renal organogenesis and cancer progression. However, their roles in kidney renal clear cell carcinoma (KIRC) remain to be elucidated.

**Methods:** FRAS1/FREM RNA expression analysis was performed using TCGA/GTEx databases, and valided using GEO databases and real-time PCR. Protein expression was peformed using CPTAC databases. Herein, we employed an array of bioinformatics methods and online databases to explore the potential oncogenic roles of FRAS1/FREM in KIRC.

**Results:** We found that FRAS1, FREM1 and FREM2 genes and proteins expression levels were significantly decreased in KIRC tissues than in normal tissues. Decreased FRAS1/FREM expression levels were significantly associated with advanced clinicopathological parameters (pathological stage, grade and tumor metastasis status). Notably, the patients with decreased FRAS1/FREM2 expression showed a high propensity for metastasis and poor prognosis. FRAS1/FREM were correlated with various immune infiltrating cells, especially CD4^+^ T cells and its corresponding subsets (Th1, Th2, Tfh and Tregs). FRAS1 and FREM2 had association with DNA methylation and their single CpG methylation levels were associated with prognosis. Moreover, FRAS1/FREM might exert antitumor effects by functioning in key oncogenic signalling pathways and metabolic pathways. Drug sensitivity analysis indicated that high FRAS1 and FREM2 expression can be a reliable predictor of targeted therapeutic drug response, highlighting the potential as anticancer drug targets.

**Conclusion:** Together, our results indicated that FRAS1/FREM family members could be potential therapeutic targets and valuable prognostic biomarkers of KIRC.

## Introduction

Renal cell carcinoma (RCC) is one of the common urinary system tumors, which affects over 400,000 individuals worldwide and its incidence continues to increase in both men and women per year (Siegel et al., 2021). There are several subvariants of RCC, the most common pathological type is kidney renal clear cell carcinoma (KIRC), which accounts for approximately 70%–80% of RCC. Although nephrectomy remains the standard care for patients with locally advanced RCC, recurrence occurs in up to 50%–80% of patients, ultimately causing death from the disease. Moreover, up to a third of KIRC at initial diagnosis will present with or develop metastases, which state is almost uniformly lethal with a poor prognosis (Diaz-Montero et al., 2020; Jonasch et al., 2021). Therefore, screening effective biomarkers for the diagnosis, treatment and prognostic evaluation of KIRC is of great clinical significance (Choueiri et al., 2022).

As one of the major components of the tumor microenvironment (TME), the extracellular matrix (ECM), which comprises proteins and polysaccharides, plays multiple crucial roles during the development of cancer (Huang et al., 2021). Moreover, the interplay between TME and malignant cells contribute to ECM stiffness, and, the stiffened ECM can also cause the changes of cancer cells (Nazemi and Rainero, 2020; Romani et al., 2021). The basement membrane (BM), a specialized type of ECM in direct contact with cells, constitutes architecturally complex ECM protein networks of great structural and regulatory importance (Stuelten et al., 2018; Reuten et al., 2021). FRAS1 (Fraser syndrome protein 1), together with FREM1 (the Fras1-related extracellular matrix proteins 1) and FREM2, belonging to the FRAS1/FREM extracellular matrix protein family, are located in the sublamina densa of basement membranes (Pavlakis et al., 2011). They can form an independent ternary complex, in which each component is necessary for stabilization of the entire complex (Pavlakis et al., 2011; Kiyozumi et al., 2012).

FRAS1, FREM1 and FREM2 are linked to human disorders. Recessive mutations in FRAS1 and FREM2 are shown to be able to cause fraser syndrome, which is a rare, hereditary, autosomal, recessive, multisystem disorder characterized by cryptophthalmos, syndactyly, renal agenesis, and a variety of morphogenetic defects (Jadeja et al., 2005; Pavlakis et al., 2011). Mutation of FREM1 can also cause another rare autosomal-recessive human disorder namely, bifid nose, renal agenesis and anorectal malformations syndrome (BNAR) (Alazami et al., 2009). Notably, the mutation of any one of FRAS1, FREM1 and FREM2 can cause congenital anomalies of the kidneys and urinary tract (CAKUT), which means that FRAS1/FREM genes and proteins play essential roles in renal organogenesis (Pavlakis et al., 2011; Al-Hamed et al., 2021). FRAS1/FREM are also found to be involved in the progression of several cancers, such as lung cancer (Zhan et al., 2014), gastric cancer (Umeda et al., 2020), breast carcinoma (Li H. N. et al., 2020) and isocitrate dehydrogenase (IDH)- wild-type glioblastoma (Jovcevska et al., 2019). However, up to now, the roles of FRAS1/FREM family in KIRC have yet been fully elucidated.

We herein conducted a comprehensive analysis to illustrate the FRAS1/FREM profiles in KIRC including expression patterns, potential functions, prognostic value, immune infiltrating levels, as well as DNA methylation levels using the TCGA project and GEO databases. Biological interaction networks and relevant cellular pathway were also analyzed to investigate the potential molecular mechanism of FRAS1/FREM in KIRC.

## Materials and methods

### Differentially expressed FRAS1/FREM at the transcriptional level

First, the mRNA expressions of FRAS1, FREM1, and FREM2 in KIRC tissues with those in normal controls were analyzed by the Oncomine database (https://www.oncomine.org, an integrated online cancer microarray database and data-mining platform), using a Student *t* test. The cut-off *p*-value and fold change were as following: *p*-value < 0.001, fold change = 1.5, gene rank = 10%.

Then TIMER 2 (tumor immune estimation resource, version 2, http://timer.cistrome.org) was used for the analysis the expression profiling of FRAS1, FREM1, and FREM2 between cancers and normal tissues. Considering that there are limited normal samples in TCGA, we have integrated data from normal tissues in the GTEx database and data from TCGA tumor tissues. RNA sequencing and related clinical data were downloaded from TCGA (http://cancergenome.nih.gov) using UCSC Xena (https://xena.ucsc.edu/), Gene expression data from normal tissues were downloaded from GTEx (https://commonfund.nih.gov/GTEx). Data analysis was conducted using R software (Version 4.0.3), and the R package “ggpubr” was used to draw box plots, *p < 0.05, **p < 0.01, ***p < 0.001).

We used the data of GEO (Gene Expression Omnibus, https://www.ncbi.nlm.nih.gov/geo) to further verify the differential expression levels of FRAS1, FREM1, and FREM2 between cancer and normal tissues in KIRC. The datasets [GSE40435 (Wozniak et al., 2013) and GSE53757 (von Roemeling et al., 2014)] used were from GEO database, and the download data format was MINIML. Box plots were drawn by boxplot; PCA graphs were drawn by R software package ggord; The box plot was implemented by the R software package ggplot2; the heat map was displayed by the R software package pheatmap.

### Differentially expressed FRAS1/FREM at protein level in KIRC

The CPTAC (Clinical proteomic tumor analysis consortium, https://proteomics.cancer.gov/programs/cptac) was used for proteomics research of various tumors. We used UALCAN tool (http://ualcan.path.uab.edu/analysisprot.html) to conduct protein expression analysis of the CPTAC dataset. Expression levels of the total protein of FRAS1, FREM1, and FREM2 had been compared between KIRC and normal tissues, respectively.

HPA (Human Protein Atlas, http://www.proteinatlas.org) is a platform that contains representative immunohistochemistry (IHC) based protein expression data for near 20 highly common kinds of cancers. To evaluate differences in FRAS/FREM protein expression, IHC images of FRAS1 and FREM2 protein expression in KIRC tissues and normal tissues, were directly visualized by HPA.

### Participants and reverse transcription PCR

The surgically resected KIRC tissue and paired normal adjacent kidney tissue were collected from 35 patients with KIRC in the department of urology in shandong provincial hospital from February to August 2022. All patients did not receive radiotherapy or chemotherapy before operation. After operation, they were pathologically diagnosed as kidney renal clear cell carcinoma and signed the informed consent. Total mRNA was isolated from frozen human tissues using the RNA-Quick Purification Kit (eSUN Bio Material Co., Ltd.) according to the manufacturer’s instruction, and mRNA levels were analyzed using real-time quantitative RT-PCR with the Bio-Rad iCycler sysytem. The sequences of the specific primers for the target genes were listed below: FRAS1, forward primer: 5′- AAT​AGC​TGC​CAA​CCA​ATG​CTG -3′, Reverse Primer: 5′- CAA​GAG​CAC​ACA​CTA​CAT​GGA​G -3′; FREM1, forward primer: 5′- GCC​TGT​GGT​AAC​CAG​GAA​CAA -3′, Reverse Primer: 5′- CGC​AGG​TGT​ATC​AGG​GTC​G -3′; FREM2, forward primer: 5′- GAG​GGG​CAG​TAG​TGC​TAC​CA -3′, Reverse Primer: 5′- GAC​CAG​AGG​CAA​GTT​CCG​A -3′; 18 s, forward primer: 5′- CGG​CGA​CGA​CCC​ATT​CGA​AC -3′, Reverse Primer: 5′- GAA​TCG​AAC​CCT​GAT​TCC​CCG​TC -3′. The data of real-time PCR were analyzed using the value 2-Δct. 18 s rRNA was used as the internal control. The continuous data were represented by Mean, SED. *p < 0.05, **p < 0.01, ***p < 0.001, and ****p < 0.0001. The paired *t* test was used to analyze the correlation between the paired tissues of the normal distribution. The wilcoxon test was used to analyze the correlation between the paired tissues of the non-normal distribution.

### Correlation between FRAS1/FREM and clinical phenotype in KIRC

Raw counts of RNA-sequencing data of FRAS1, FREM1 and FREM2 and corresponding clinical information such as pathological stage, grade and prognosis from 530 KIRC samples were obtained from TCGA. Sanguini diagram was built based on the R software package ggalluval. All the above analysis methods and R package were implemented by R foundation. *p* < 0.05.

The expression distribution of FRAS1, FREM1 and FREM2 genes in different pathological stage (stage I, stage II, stage III and stage IV and normal tissues), different pathological grade (grade I, grade II, grade III and grade IV and normal tissues) and different state (with and without metastasis) were implemented by R foundation and ggplot2 (v3.3.2). *p < 0.05, **p < 0.01, ***p < 0.001. When the number of groups greater than or equal to 3, Kruskal–Wallis test was used, otherwise, Wilcoxon test was used.

We used GEO database to further verify the expression distribution of FRAS1, FREM1 and FREM2 genes in different pathological stage (Stage I + II, Stage III + IV) and different pathological grade (Grade I + II, Grade III + IV) in KIRC. The pathological stage data was from GSE73731 and GSE53757, while, the pathological grade was from GSE 40435 and GSE73731. Box plots are drawn by boxplot; PCA graphs are drawn by R software package ggord; the box plot was implemented by the R software package ggplot2.

The UALCAN tool was also applied to analyse the correlation between FRAS1/FREM proteins expression and pathological stage and grade with the date from CPTAC.

As the TCGA dateset didn’t include metastatic tumor tissues, we also used GEO database [GSE105261 (Nam et al., 2019) and GSE22541 (Wuttig et al., 2012)] to compare the expression distribution of FRAS1, FREM1 and FREM2 genes in primary KIRC and metastatic KIRC. What’s more, we further divided the patients from GSE22541 in three groups: The low matestasis risk primary RCC (*n* = 16, eight patients with distant or lymph node metastases within one year after the nephrectomy were excluded); pulmonary metastasis of KIRC, No. (the number of matastases) < 10; pulmonary metastasis of KIRC, No. (the number of matastases) > = 10. The differential expression of FRAS1, FREM1 and FREM2 genes in these groups were compared. It is worth noting that, GSE155209 was used to verify the diagnostic value of FRAS1, FREM1 and FREM2 genes for metastatic progression in stage I and stage II KIRC. Box plots were drawn by boxplot; the box plot was implemented by the R software package ggplot2. *p < 0.05, **p < 0.01, ***p < 0.001, ****p < 0.0001. When the number of groups greater than or equal to 3, Kruskal–Wallis test was used, otherwise, Wilcoxon test was used.

### Correlation between FRAS1/FREM and survival analysis in KIRC

Raw counts of RNA-sequencing data of the FRAS1, FREM1 and FREM2 and corresponding clinical information from 530 KIRC patients were obtained from TCGA. We performed survival analysis to search for relationships between gene expression and patient prognosis, such as OS, DSS and PFS, computed the log-rank *p* value and hazard ratio (HR) with 95% confidence intervals (95% CI) using “survival” package in R. The results were displayed as forestplots (using “forestplot” package in R) and survival curves. For Kaplan–Meier curves, *p*-values and HR with 95% CI were generated by log-rank tests and univariate Cox proportional hazards regression.

Multivariate cox regression analysis was performed to evaluate the utility of FRAS1, FREM1 and FREM2 expression in predicting cancer patient prognosis. The forest was used to show the *p* value, HR and 95% CI of each variable through “forestplot” R package. HR and *p* value of constituents involved in multivariate Cox regression and some parameters (age, gender, race, TNM stage and grade).

### Correlation analysis between FRAS1/FREM and immune infiltrating levels

Pearson analysis was performed to assess the correlations between FRAS1/FREM gene expression and quantitative variables such as immune checkpoints, TMB, MSI as well as MMR proteins. A *p*-value of less than 0.05 was considered statistically significant. The results were displayed as heatmaps using “pheatmap” package in R.

The relationship between FRAS1/FREM gene expression and Tumor-infiltrating immune cell profiles across TCGA pan-cancer cohort was analyzed by TIMER 2. The TIMER, TIDE, CIBERSORT, CIBERSORT-ABS, QUANTISEQ, XCELL, MCPCOUNTER and EPIC algorithms were applied for estimations. Furthermore, the immune cell subsets associated with CD4^+^ T cells including Th1, Th2, T cell follicular helper, T cell regulatory (Tregs) were selected for detailed analysis.

### Correlation between FRAS1/FREM expression and DNA methylation

The UALCAN tool was applied to analyse the correlation between FRAS1/FREM gene expression and the gene promoter methylation level for KIRC. Then we analysed the assciation between FRAS1/FREM gene expression and four DNA methyltransferases (DNMT1: red, DNMT2: blue, DNMT3A: green, DNMT3B: purple) for each tumor, and visualization were performed as follows.

MethSurv (https://biit.cs.ut.ee/methsurv/), a web tool for survival analysis based on CpG methylation patterns, was applied to explore the prognostic value of single CpG methylation of FRAS1 and FREM2 in KIRC patients (*p* < 0.05 as significant).

### Function enrichment of FRAS1/FREM in KIRC

GeneMANIA 3.6.0 (http://www.genemania.org) is a website for generating hypotheses about gene function using available genomics and proteomics data. In our study, the FRAS1, FREM1 and FREM2 genes were submitted to the GeneMANIA to illustrate the functional association network among FRAS1/FREM and their related genes. Protein–protein interaction (PPI) network construction Search Tool for the Retrieval of Interacting Genes (STRING; http://string-db.org) online database was used to predict PPI network of co-regulated hub genes and analyse the functional interactions between proteins.

Gene set enrichment analysis (GSEA) was performed to explore the potential mechanisms involved in the effect of risk score on KIRC using transcriptional sequences in TCGA database. The enrichment analysis was performed using the Molecular Signatures Database (MSigDB) of H (hallmark), C2 (C2:CP:KEGG) and C5 (C5:GO:BP, CC, MF). The enriched gene sets in the GSEA that reached a nominal significance level of *p* < 0.05 and normalized Enrichment Score (NES) > 1.5 were considered significant. The top 20 sets of each phenotype associated with genes were listed to reflect the role of FRAS1, FREM1 and FREM2.

### Drug sensitivity analysis

The data of gene expression level and corresponding clinical information were downloaded from the TCGA dataset. The largest publicly available pharmacogenomics database [the Genomics of Drug Sensitivity in Cancer (GDSC), https://www.cancerrxgene.org/] was used to predict the targeted therapeutic response for each sample. The prediction process was implemented by R package “pRRopheticm”. The samples’ half-maximal inhibitory concentration (IC50) was estimated by ridge regression. Spearman’s-correlation analysis was used to explore the correlation between drug sensitivity and FRAS1, FREM1 and FREM2 gene expression in KIRC.

## Results

### Transcriptional levels and protein levels of FRAS1/FREM in KIRC

**FIGURE 1:**
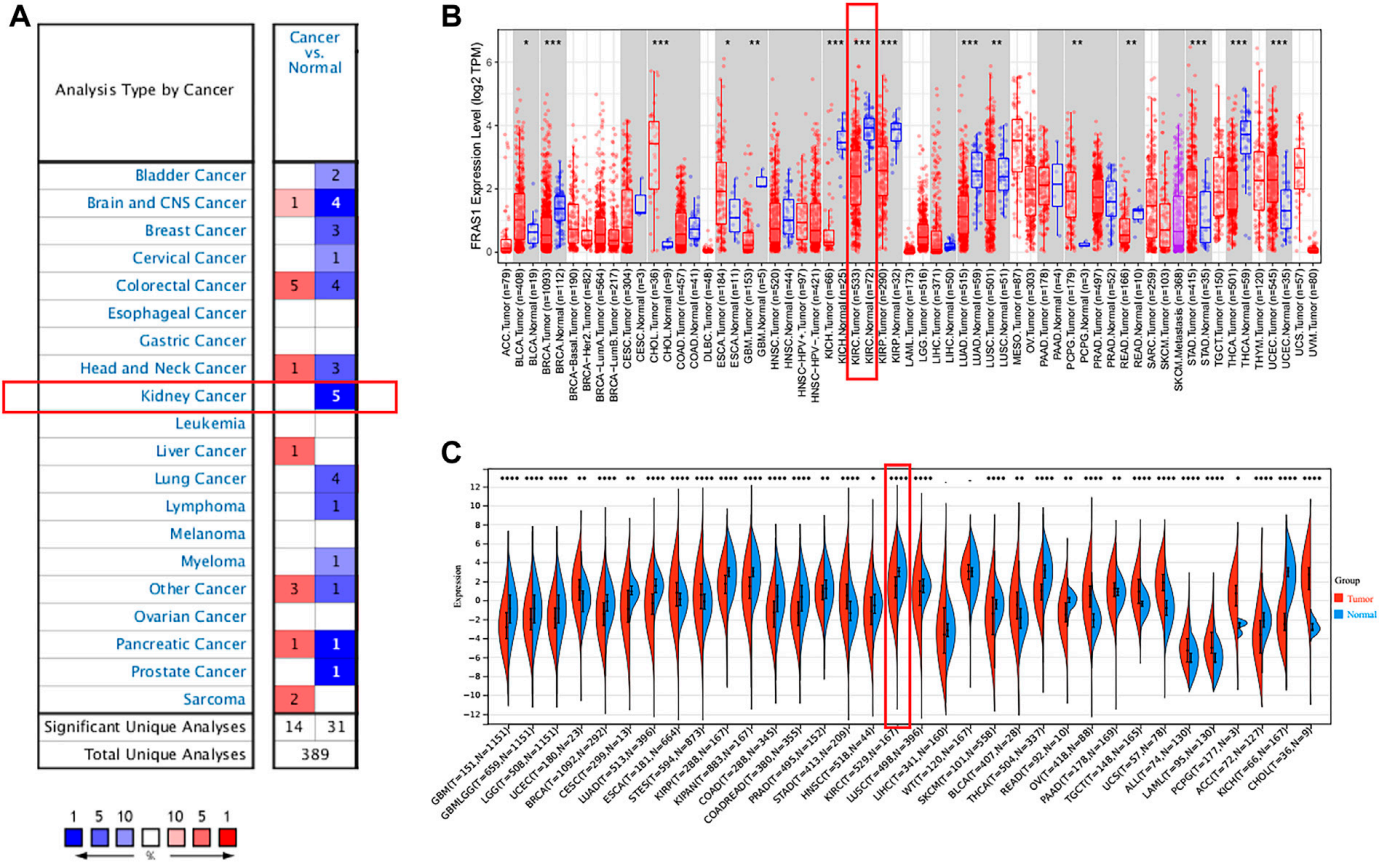
Pan-cancer expression analysis of FRAS1 mRNA in human tumors vs. normal tissues. **(A)** Differential expression of FRAS1 in the Oncomine database. **(B)** The expression profile of FRAS1 in pan-cancer analysis by TIMER2.0. **(C)** The expression profile of FRAS1 in pan-cancer analysis from TCGA database and GTEx database. (*p < 0.05, **p < 0.01, ***p < 0.001).

The mRNA expression levels of FRAS1, FREM1 and FREM2 were analyzed in Oncomine over a cancer-wide range. As shown in Figure 1A, Supplementary Figure S1A,2A, FRAS1 expression was lower in most cancer groups compared with the respective normal groups. The mRNA expression levels of FRAS1, FREM1 and FREM2 were significantly downregulated in KIRC, the detailed results were summarized in Table 1. The mRNA expression level of FRAS1 was significantly downregulated in patients with KIRC in three datasets (with a fold change of -3.812 in Higgins Renal dateset, -5.118 in Yusenko Renal dateset and -2.205 in Beroukhim Renal dateset). Similarly, the mRNA expression level of FREM1 was also downregulated in patients with KIRC in two datasets (with a fold change of -1.664 in Lenburg Renal dateset and -20.647 in Yusenko Renal dateset). The mRNA expression level of FREM2 was also downregulated with a fold change of -5.168 in Yusenko Renal dateset.

**TABLE 1:** Differential expression of FRAS1/FREM family members in KIRC tissues and normal kidney tissues in the Oncomine database.

	Types of KIRC VS. Normal	Fold change	*p*-value	*t*-test	Ref
FRAS1					
	Clear Cell Renal Cell Carcinoma	-3.812	2.98E-05	-7.000	Higgins Renal
	Clear Cell Renal Cell Carcinoma	-5.118	6.19E-05	-4.933	Yusenko Renal
	Non-Hereditary Clear Cell Renal Cell Carcinoma	-2.205	5.10E-05	-5.495	Beroukhim Renal
FREM1					
	Clear Cell Renal Cell Carcinoma	-1.664	2.35E-06	-7.279	Lenburg Renal
	Clear Cell Renal Cell Carcinoma	-20.647	2.05E-04	-6.737	Yusenko Renal
FREM2					
	Clear Cell Renal Cell Carcinoma	-5.168	1.27E-04	-4.257	Yusenko Renal

Next, we compared the FRAS1, FREM1 and FREM2 expression levels across all TCGA tumors using TIMER 2.0 (as shown in Figure 1B; Supplementary Figures S1B, 2B). Considering the number size of normal tissue in the TCGA database is small, we further matched the GTEx normal tissues with the TCGA cancer tissues to reflect the gene expression landscape in a more convincing manner (as shown in Figure 1C; Supplementary Figures S1C, 2C). Consistent from the Oncomine database, the mRNA expression levels of FRAS1, FREM1 and FREM2 were still significantly down-regulated in KIRC tissues compared to normal samples (*p* < 0.001).

**FIGURE 2:**
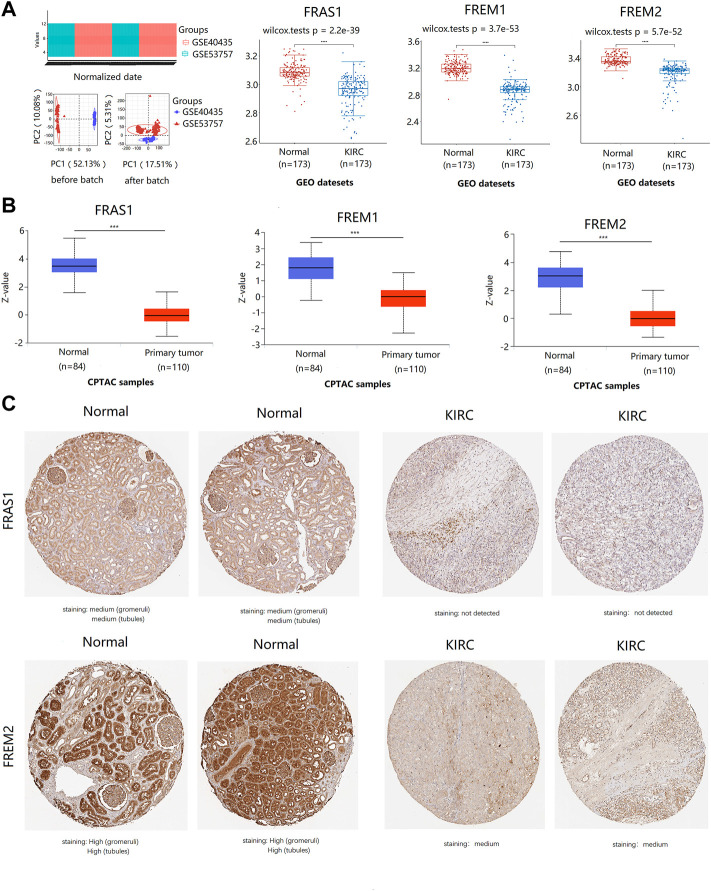
The expression profile of FRAS1/FREM family members in KIRC tissues and normal kidney tissues with datesets from GEO and CPTAC datebase. **(A)** the expression profile of FRAS1/FREM mRNA in KIRC samples and paired normal samples with datesets from the GEO database (GSE40435 and GSE53757) (****p < 0.0001). **(B)** Protein expression of FRAS1/FREM in KIRC tissues and normal kidney tissues with datesets from CPTAC datebase. ***p < 0.001. **(C)** Representative immunohistochemistry images of FRAS1 and FREM2 in normal kidney tissues and KIRC tissues (HPA database).

Furthermore, we used datasets GSE40435 (Wozniak et al., 2013) (101 KIRC vs. 101 normal) and GSE53757 (von Roemeling et al., 2014) (72 KIRC vs. 72 normal) from GEO database to further validate this conclusion, As shown in Figure 2A, the mRNA expression levels of FRAS1, FREM1 and FREM2 were also significantly downregulated in the 173 KIRC tissues included compared to matched normal tissues (*p* < 0.0001).

In addition to transcription, we explored the protein expression of FRAS/FREM family members in KIRC by CPTAC and the Human Protein Atlas. The protein expression levels of FRAS1, FREM1 and FREM2 were lower in KIRC tissues than in normal tissues using the CPTAC dataset (Figure 2B, *p* < 0.001). Similar results appeared by CPTAC analysis, We found that KIRC tissues had negative or medium IHC staining, while normal kidney tissues had medium or high staining by HPA. Negative expression of FRAS1 protein was observed in KIRC tissues, while medium protein expression was observed in normal kidney tissues. Medium protein expression of FREM2 was observed in KIRC tissues, while high protein expression was observed in normal kidney tissues (Figure 2C).

**FIGURE 3:**
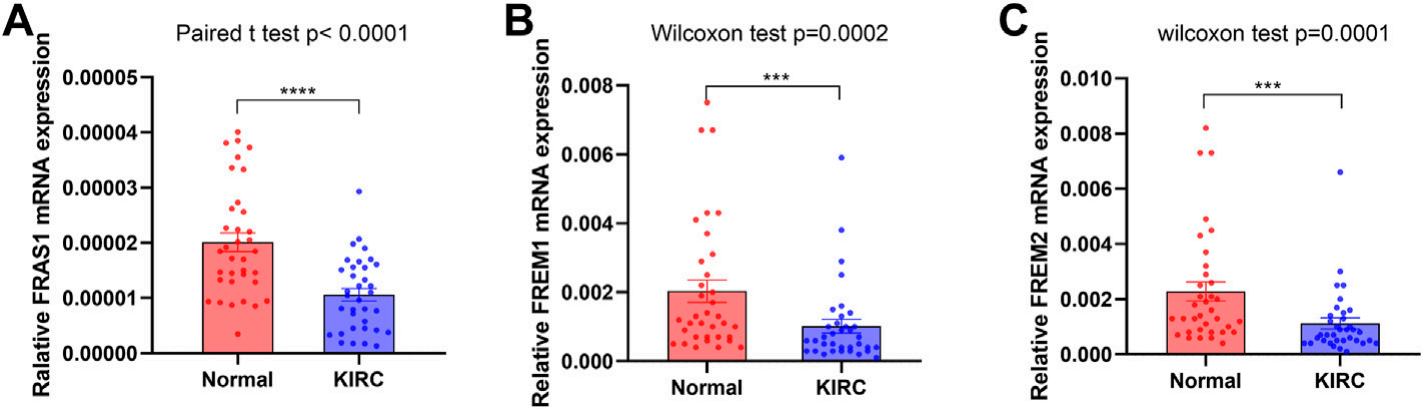
FRAS1/FREM expression in cancer and adjacent normal kidney tissues of KIRC patients by RT-PCR. **(A)**FRAS1 expression in cancer and adjacent normal kidney tissues of KIRC patients. **(B)** FREM1 expression in cancer and adjacent normal kidney tissues of KIRC patients. **(C)** FREM2 expression in cancer and adjacent normal kidney tissues of KIRC patients.

The expression levels of FRAS1, FREM1 and FREM2 in both KIRC and non-tumor tissues were also measured by qRT-PCR verification, which confirmed the expression profiles of FRAS1/FREM family. Compared with normal tissues, the expression levels of FRAS1 (Figure 3A, *p* < 0.0001), FREM1 (Figure 3B, *p* = 0.0002) and FREM2 (Figure 3C, *p* = 0.0001) were significantly decreased in KIRC tissues.

Taken together, all the results above showed that the expression levels of FRAS1, FREM1 and FREM2 were downregulated in KIRC both in the transcriptional and protein expressions.

### Correlation of FRAS1/FREM expression with clinical phenotypes in KIRC

**FIGURE 4:**
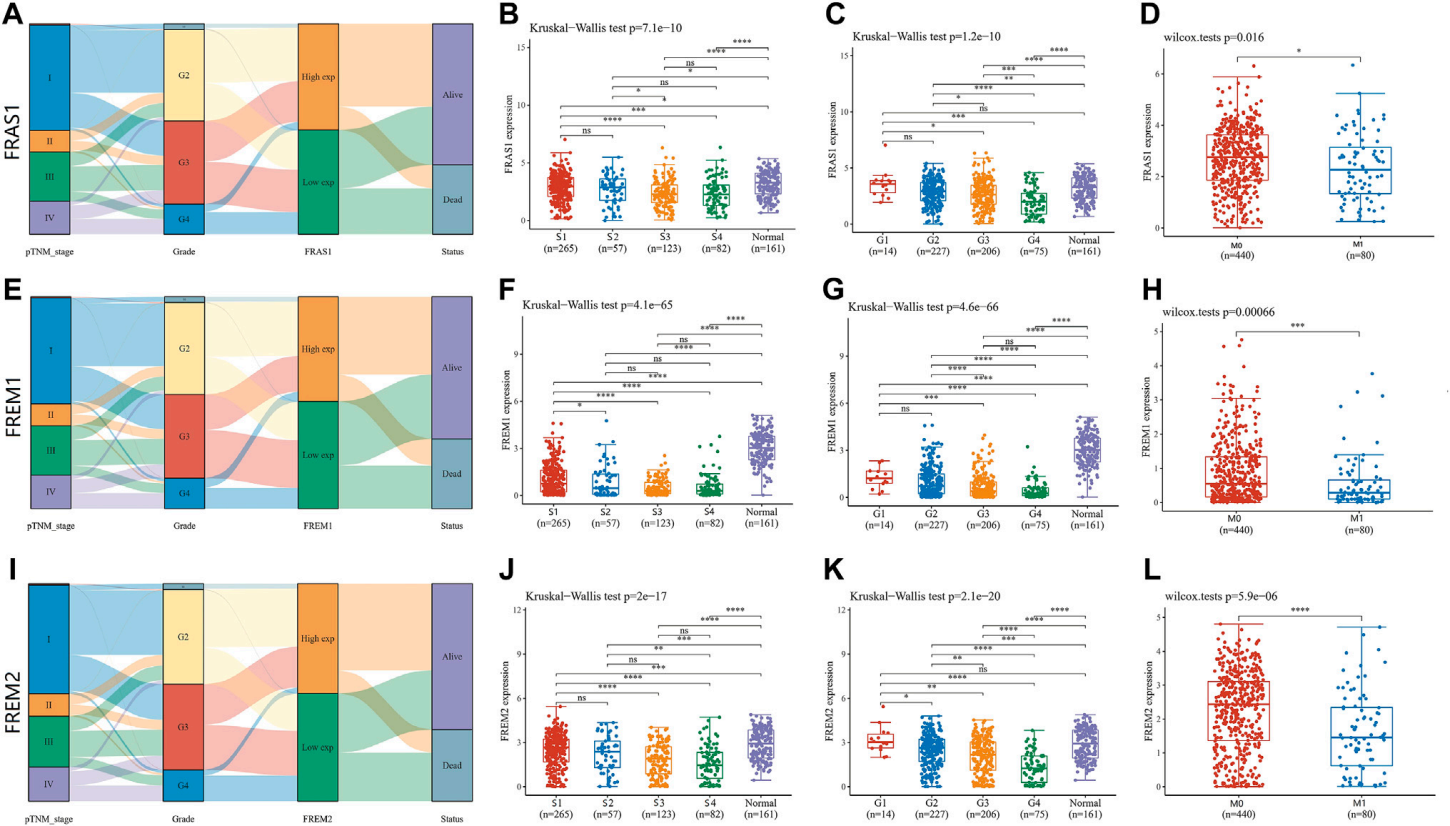
Correlation of FRAS1/FREM mRNA expression with clinicopathological parameters (such as pathological stage, grade, tumor metastasis status and prognosis) in KIRC with datesets from TCGA datebase (*p < 0.05, **p < 0.01, ***p < 0.001, ****p < 0.0001). **(A)**Sanguini diagram of the relevance of FRAS1 and corresponding clinical information. **(B)** The relevance of FRAS1 and pathological stage. **(C)** The relevance of FRAS1 and pathological grade. **(D)** The relevance of FRAS1 and tumor metastasis status. **(E)** Sanguini diagram of the relevance of FREM1 and corresponding clinical information. **(F)** The relevance of FREM1 and pathological stage. **(G)** The relevance of FREM1 and pathological grade. **(H)** The relevance of FREM1 and tumor metastasis status. **(I)** Sanguini diagram of the relevance of FREM2 and corresponding clinical information. **(J)** The relevance of FREM2 and pathological stage. **(K)** The relevance of FREM2 and pathological grade. **(L)** The relevance of FREM2 and tumor metastasis status.

Sanguini diagram was built to show the relevance of FRAS1/FREM expression levels and corresponding clinical information such as pathological stage, grade and prognosis. Notably, we found a significant correlation between them, as KIRC progressed, the low expression levels of FRAS1, FREM1, FREM2 were associated with the advanced pathological stage, the advanced pathological grade and poor prognosis (Figures 4A,E,I). Next, we verified this tendency by Kruskal–Wallis test and Wilcoxon test. The low expression level of FRAS1 gene was significantly correlated with advanced clinical stage (*p* = 7.1E-10, Figure 4C), high pathological grade (*p* = 1.2E-10, Figure 4D) and tumor metastasis status (*p* = 0.016, Figure 4E). The low expression level of FREM1 gene was significantly correlated with advanced clinical stage (*p* = 4.1E-65, Figure 4F), high pathological grade (*p* = 4.6E-66, Figure 4G) and tumor metastasis status (*p* = 0.00066, Figure 4H). The low expression level of FREM2 gene was also significantly correlated with advanced pathological stage (p = 2E-17, Figure 4J), high pathological grade (*p* = 2.1E-20, Figure 4K) and tumor metastasis status (*p* = 5.9E-06, Figure 4L).

**FIGURE 5:**
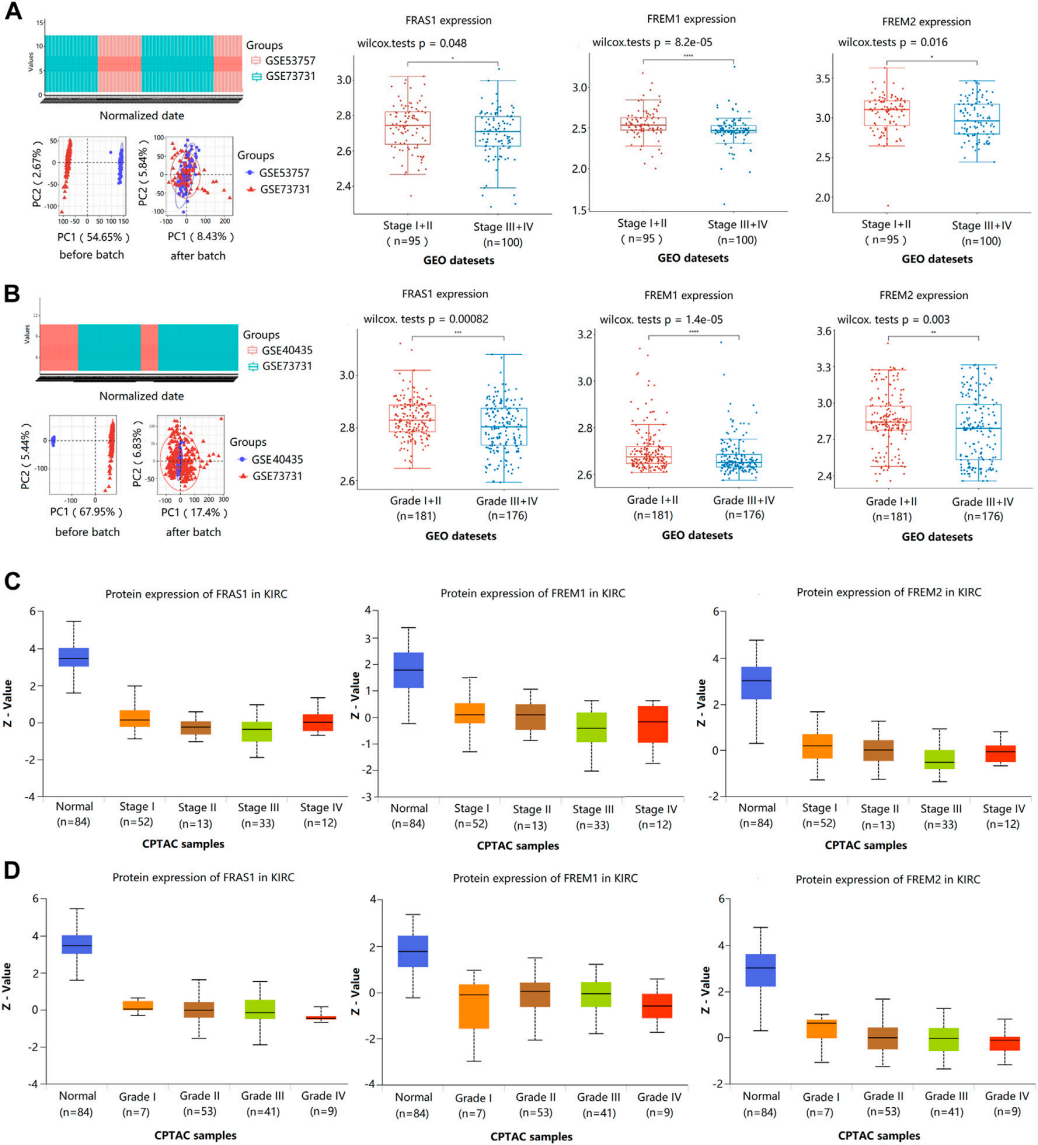
Correlation of FRAS1/FREM mRNA expression with clinical phenotypes in KIRC with datesets from GEO and CPTAC datebase. **(A)** Correlation of FRAS1/FREM mRNA expression with pathological stage in KIRC with datesets from GEO datebase (GSE53757 and GSE73731) (*p < 0.05, ****p < 0.0001). **(B)** Correlation of FRAS1/FREM expression with pathological grade in KIRC with datesets from GEO datebase (GSE40435 and GSE73731) (*p < 0.05, ****p < 0.0001). **(C)** Correlation of FRAS1/FREM protein expression with pathological stage in KIRC with datesets from CPTAC datebase. **(D)** Correlation of FRAS1/FREM protein expression with pathological grade in KIRC with datesets from CPTAC datebase.

Furthermore, we validated the association of FRAS1/FREM mRNA expression levels and pathological stage using GSE53757 (43 Stage I + II vs. 29 Stage III + IV) and GSE73731 (Wei et al., 2017) (52 Stage I + II vs. 71 Stage III + IV). We found that the low expression levels of FRAS1 (*p* = 0.048), FREM1 (*p* = 8.2E-05), and FREM2 (*p* = 0.016) mRNA expression levels were significantly correlated with advanced pathological stage (Figure 5A). Then, we validated the association of FRAS1/FREM expression levels and pathological grade using GSE40435 (69 Grade I + II vs. 32 Grade III + IV) and GSE73731 (112 Grade I + II vs. 144 Grade III + IV). We also found that the low expression levels of FRAS1 (*p* = 0.00082), FREM1 (*p* = 1.4E-05), and FREM2 (*p* = 0.003) mRNA expression levels were significantly correlated with advanced pathological grade (Figure 5B). The low expression levels of FRAS1, FREM1 and FREM2 proteins were also correlated with advanced pathological stage and pathological grade by CPTAC, due to the limited sample size (Figure 5C,D). These data above indicated that FRAS1, FREM1 and FREM2 played significant roles in the progression of KIRC.

**FIGURE 6:**
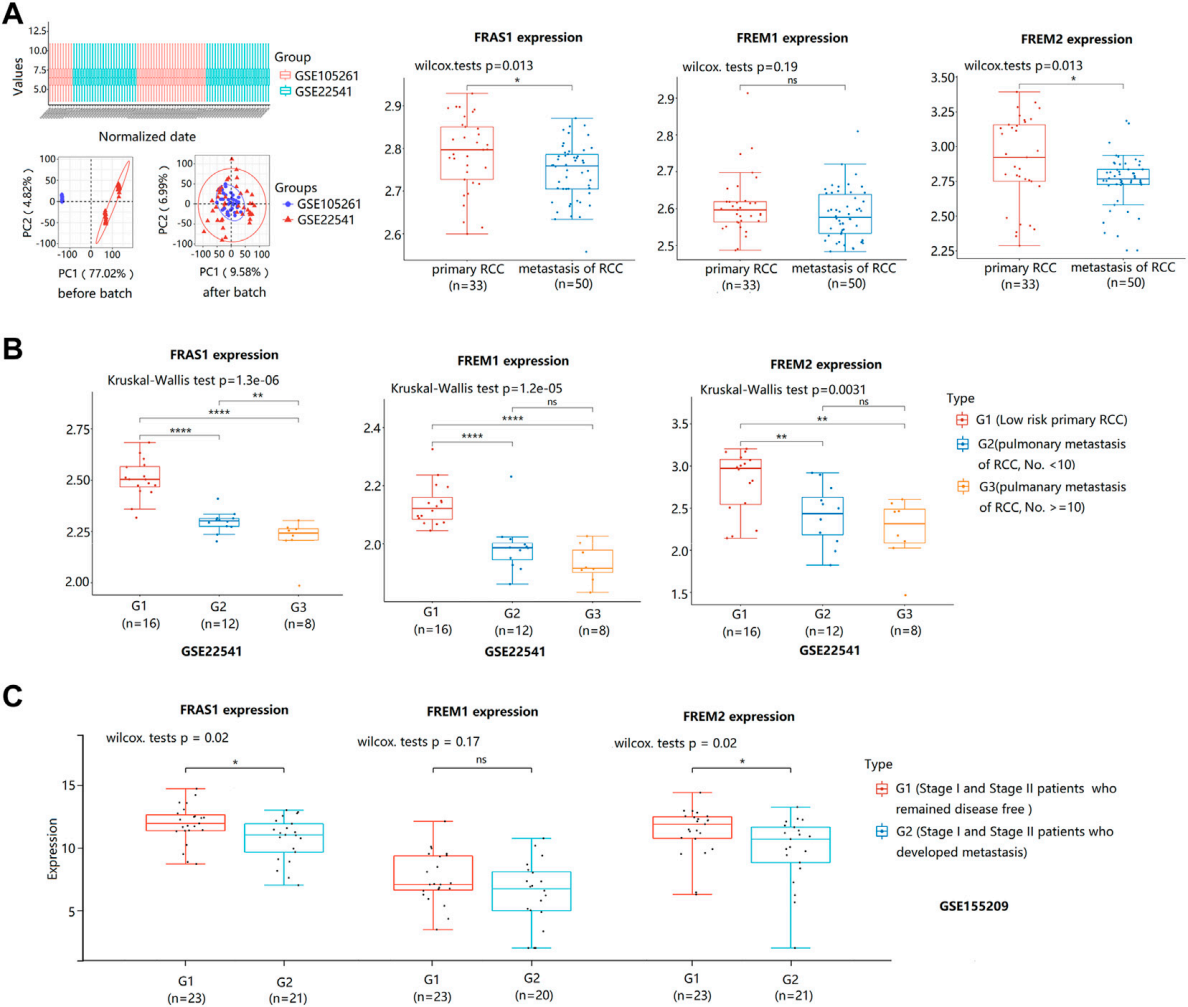
FRAS1/FREM mRNA expression between primary KIRC and matestasis of KIRC with datesets from GEO datebase. **(A)**FRAS1/FREM mRNA expression between primary KIRC and matestasis of KIRC with datesets from GEO datebase (GSE105261 and GSE22541) (*p < 0.05, ns, not statistically significant). **(B)** FRAS1/FREM mRNA expression in three groups: The low matestasis risk primary RCC (*n* = 16), pulmonary metastasis of KIRC, the number of matastases <10 (*n* = 12), pulmonary metastasis of KIRC, the number of matastases > = 10 (*n* = 8) in KIRC from GSE22541. (**p < 0.01, ****p < 0.0001). **(C)** FRAS1/FREM mRNA expression between stage I and stage II patients who developed metastasis within 5 years of nephrectomy and patients who remained disease free (*p < 0.05, ns, not statistically significant).

Notably, we used datasets GSE105261 (9 primary KIRC vs. 26 matestasis of KIRC) and GSE22541 (24 primary KIRC vs. 24 matestasis of KIRC) from GEO database to study whether FRAS1/FREM could be tumor metastasis markers. As shown in Figure 6A, the mRNA expression levels of FRAS1 and FREM2 were also significantly downregulated in the matestasis of KIRC tissues included compared to matched normal tissues (*p* < 0.05). What’s more, the expression level of FRAS1 was significantly correlated with the number of pulmonary matastases (Figure 6B). Interesting that FRAS1 and FREM2 expression decreased in 23 stage I and stage II patients who developed metastasis within 5 years of nephrectomy, compared to 21 patients who remained disease free (*p* < 0.05, Figure 6C).

### Correlation of FRAS1/FREM expression with patient prognosis in KIRC

**FIGURE 7:**
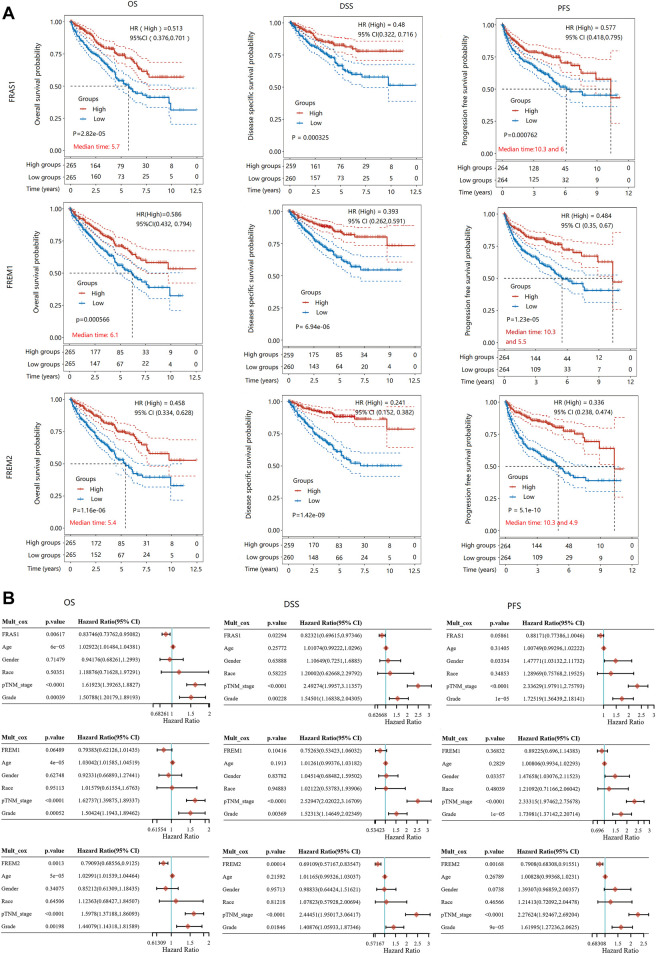
The prognostic value of mRNA level of FRAS1/FREM family members in KIRC patients. **(A)** Survival curves comparing the high and low expression of FRAS1, FREM1 and FREM2. (Kaplan-Meier survival analysis). **(B)** Hazard ratio and *p* value of constituents involved in multivariate Cox regression adjusted for clinical parameters (age, gender, race, TNM stage and grade).

To further evaluate the value of FRAS1/FREM in predicting the prognosis of KIRC cancer patients, the association between FRAS1, FREM1 and FREM2 expression and overall survival (OS), disease-specific survival (DSS) and progression-free survival (PFS) was analyzed using TCGA. Elevated FRAS1 expression was significantly related to a better OS (HR = 0.513, 95% CI = 0.376–0.701, *p* = 2.82E-05), DSS (HR = 0.48, 95% CI = 0.322–0.716, *p* = 0.000325) and PFS (HR = 0.577, 95% CI = 0.418–0.795, *p* = 0.000762) in KIRC. Similarly, high expression of FREM1 was significantly associated with a better OS (HR = 0.586, 95% CI = 0.432–0.794, *p* = 0.000566), DSS (HR = 0.393, 95% CI = 0.262–0.591, *p* = 6.94E-06) and PFS (HR = 0.484, 95% CI = 0.35–0.67, *p* = 1.23E-05) in KIRC. High expression of FREM2 was also significantly associated with a better OS (HR = 0.458, 95% CI = 0.334–0.628, *p* = 1.16E-06), DSS (HR = 0.241, 95% CI = 0.152–0.382, *p* = 1.42E-09) and PFS (HR = 0.336, 95% CI = 0.238–0.474, *p* = 5.1E-10) in KIRC. The survival curves were displayed as Figure 7A.

As above, pathological stage and grade were assiociated with FRAS1/FREM expression levels and were also highly associated with prognosis. As shown in Figure 7B, multivariate Cox proportional-hazards regression adjusted for clinical parameters (age, gender, race, TNM stage and grade) still suggested that FRAS1 expression was an independent risk factor for better prognosis (OS: HR = 0.83746, 95% CI = 0.73762–0.95082, *p* = 0.00617; DSS: HR = 0.82321, 95% CI = 0.69615–0.97346, *p* = 0.02294; PFS: HR = 0.88171, 95% CI = 0.77386–1.0046, *p* = 0.05861). FREM2 expression was also an independent risk factor for better prognosis (OS: HR = 0.79093, 95% CI = 0.68556–0.9125, *p* = 0.0013; DSS: HR = 0.69109, 95% CI = 0.57167–0.83547; PFS: HR = 0.7908, 95% CI = 0.68308–0.91551, *p* = 0.00168).

### Correlation of FRAS1/FREM expression with immune infiltrating levels in KIRC

Mismatch repair (MMR), microsatellite instability (MSI) and tumor mutation burden (TMB) are responsible for tumor initiation and regard as independent predictors of immune checkpoint blockade efficacy (Baretti and Le, 2018; Jardim et al., 2021). Here we examined the correlation between FRAS1/FREM expression and several essential MMR signatures. As shown in Supplementary Figure S3A, FRAS1 expression was positively correlated with MutL homolog 1 (MLH1), MutS homolog 2 (MSH2), MutS homolog 6 (MSH6) and PMS1 homolog 2 (PMS2) in KIRC. FREM1 expression was positively correlated with MLH1, PMS2 and epithelial cell adhesion molecule (EpCAM) in KIRC (Supplementary Figure S3B). FREM2 expression was positively correlated with MLH1, MSH2, MSH6, PMS2 and EpCAM in KIRC (Supplementary Figure S3C). In addition, as shown in Supplementary Figure S4, FRAS1 and FREM2 expression were positively correlated with MSI (*p* = 6E-04 and *p* = 0.024, respectively), while FREM1 expression was negatively correlated with TMB (*p* = 2E-05) in KIRC.

**FIGURE 8:**
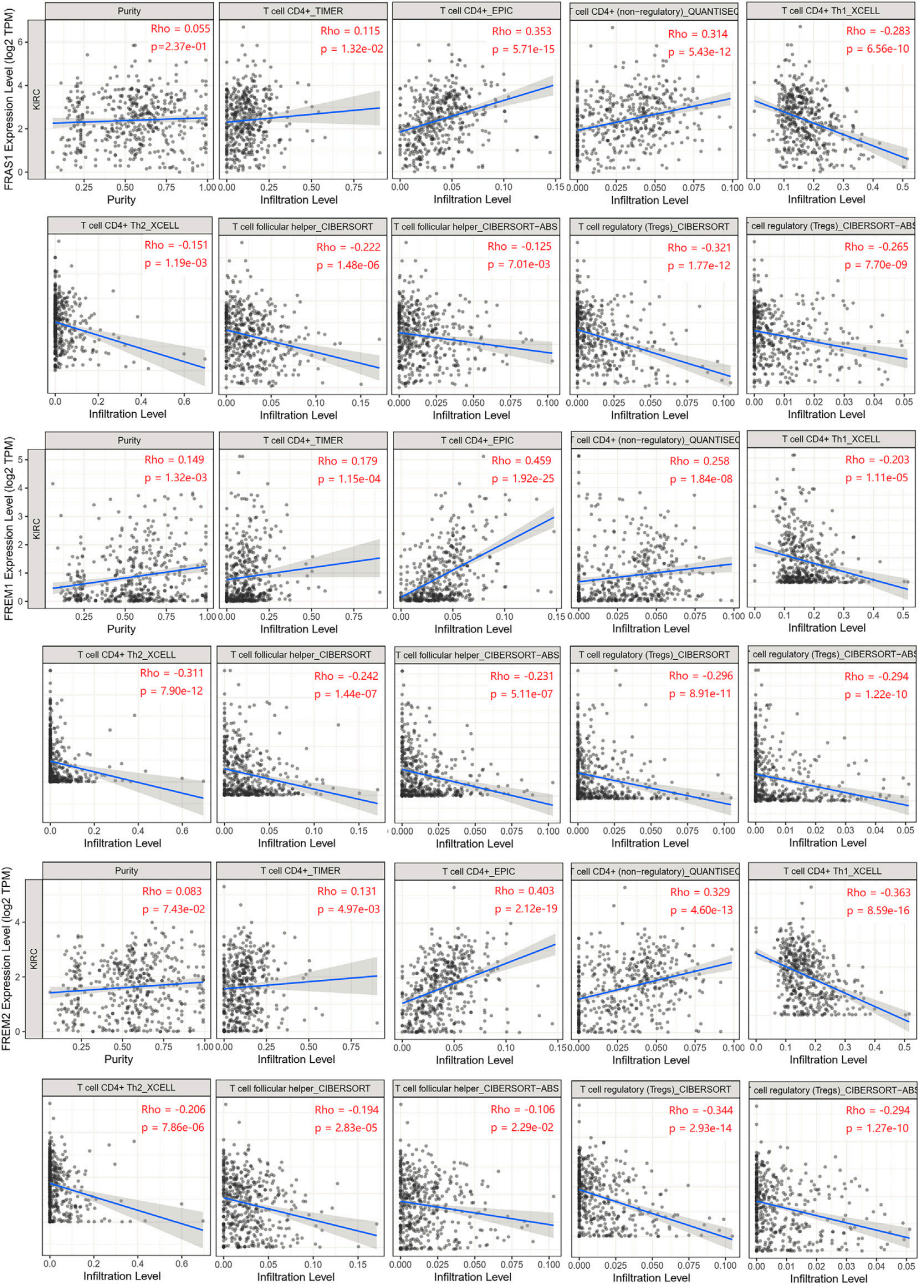
Relationship between FRAS1/FREM expression and CD4^+^ T cells, its corresponding subsets (Th1, Th2, Tfh, and Tregs) in KIRC.

To explore whether FRAS1, FREM1 and FREM2 were involved in the process of immune infiltration in KIRC, we employed TIMER 2.0 to exhibit the landscape of FRAS1/FREM correlating with tumor purity and various immune infiltrates in human cancers. As Supplementary Figure S5 indicated, the expression levels of FRAS1, FREM1 and FREM2 were positively correlated with immune infiltrating levels of multiple infiltrates including CD4^+^ T cells (*p* < 0.05) and macrophages (*p* < 0.05). Th1, Th2, induced or natural regulatory T cells (iTregs and nTregs), and Tfh (T cell follicular helper) cells are the main CD4^+^ T cell subsets (Oh and Fong, 2021). Herein, we used the TIMER, CIBERSORT, CIBERSORT-ABS, QUANTISEQ, XCELL, MCPCOUNTER and EPIC algorithms to investigate the potential relationship between the infiltration level of CD4^+^ T cells subsets and FRAS1/FREM gene expression in KIRC. The FRAS1 expression level in KIRC is positively correlated with the infiltration level of CD4^+^ T cells and negatively correlated with the infiltration level of Th1, Tfh and Tregs. Likewise, the same trend goes as FREM1 and FREM2 unfolds (Figure 8).

### Correlation between FRAS1/FREM expression and DNA methylation

**FIGURE 9:**
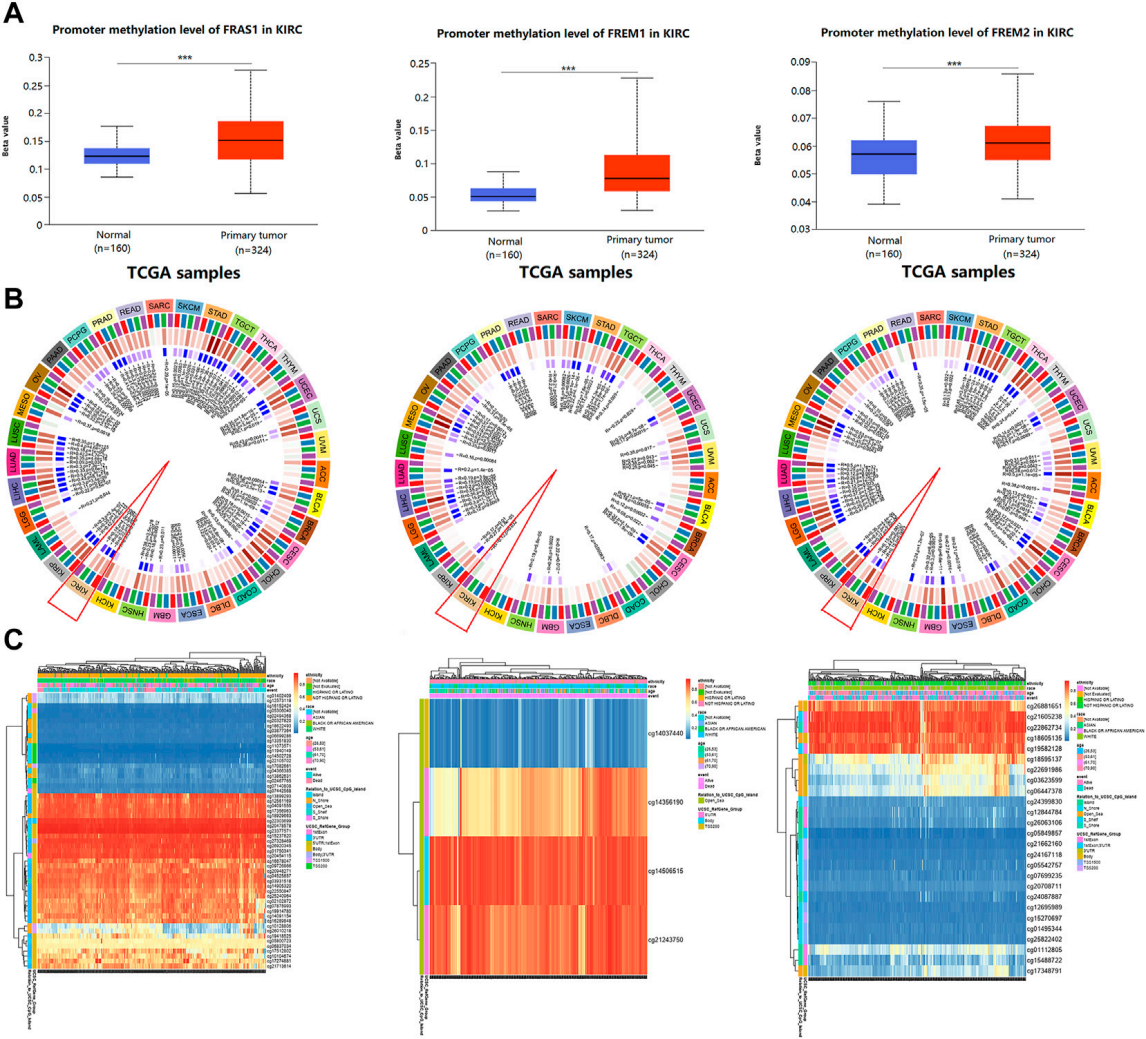
The correlation between FRAS/FREM expression and DNA methylation. **(A)** The promoter methylation levels of FRAS1, FREM1 and FREM2 in KIRC tissues vs. normal tissues. **(B)** The correlations between FRAS1, FREM1, FREM2 expression and three DNA methyltransferases (DNMT1, DNMT2 and DNMT3A). **(C)** The heat map of DNA methylation of FRAS1, FREM1, FREM2 in KIRC.

DNA methylation has been recognized as an important biological process of tumorigenesis and cancer development (Koch et al., 2018). We calculated the levels of correlation between FRAS1/FREM gene expression and their promoter methylation using UALCAN. As Figure 9A indicated, the promoter methylation levels of FRAS1, FREM1 and FREM2 were significantly higher in KIRC than normal tissues (*p* < 0.001). Moreover, there were positive correlations between FRAS1/FREM2 expression and three DNA methyltransferases (DNMT1, DNMT2 and DNMT3A) (Figure 9B). The heat map of DNA methylation results of FRAS1, FREM1 and FREM2 in KIRC via the MethSurv platform were displayed in Figure 9C. Further, we conducted Kaplan-Meier survival analysis based on CpG methylation patterns to explore the prognostic value of single CpG methylation of FRAS1 and FREM2. We found that single CpG methylation levels of FRAS1 and FREM2 were associated with prognosis in KIRC. Among them, the top ten significant prognostic values of FRAS1 and FREM2 single CpG methylation were displayed in Supplementary Figure S6 and summarized in Table 2.

**TABLE 2:** The prognostic value of single CpG of FRAS1 and FREM2 in KIRC by MethSurv (*p* < 0.05).

Gene	Relation to island	Genomic region	CpG site	Best_split	HR	CI	LR test *p* value
FRAS1							
	Open_Sea	Body	cg20948271	q25	0.41	(0.276; 0.611)	2.95E-05
	Open_Sea	Body	cg17512,802	mean	2.544	(1.65; 3.922)	7.49E-06
	Island	Body	cg02494368	mean	0.441	(0.286; 0.681)	0.000104156
	Open_Sea	Body	cg14091154	q25	0.47	(0.314; 0.704)	0.000486185
	Open_Sea	Body	cg04091555	q25	0.51	(0.341; 0.764)	0.001728597
	N_Shore	Body	cg03877364	median	1.856	(1.253; 2.75)	0.001735773
	Island	TSS200	cg11073571	q75	0.423	(0.244; 0.733)	0.000734126
	Island	TSS200	cg11940149	q75	0.428	(0.247; 0.741)	0.000872463
	Island	Body	cg16678047	q25	2.328	(1.341; 4.041)	0.000980456
	Island	Body	cg04366385	q75	0.435	(0.251; 0.752)	0.001079704
FREM2							
	Open_Sea	Body	cg26881651	mean	0.489	(0.333; 0.719)	0.000342
	Open_Sea	3′UTR	cg18595137	q25	2.426	(1.38; 4.265)	0.000642
	N_Shore	TSS1500	cg05542757	q75	1.877	(1.257; 2.804)	0.003037
	Island	1stExon	cg12844784	q25	2.383	(1.306; 4.348)	0.001604
	Open_Sea	Body	cg03623599	q25	1.926	(1.171; 3.17)	0.00591
	N_Shore	TSS200	cg21662160	q75	1.607	(1.067; 2.419)	0.02758
	Island	1stExon	cg26063106	q75	1.597	(1.063; 2.398)	0.028575
	N_Shore	TSS1500	cg25822402	q75	0.575	(0.352; 0.941)	0.020161
	S_Shelf	1stExon	cg19582,128	mean	0.647	(0.436; 0.96)	0.034219
	Island	1stExon; 5′UTR	cg24087887	q25	1.668	(1.024; 2.715)	0.030612

Abbreviations: KIRC, kidney renal clear cell carcinoma; HR, hazard ratio; LR, test, likelihoodratio test.

### Function enrichment of FRAS1/FREM in KIRC

**FIGURE 10:**
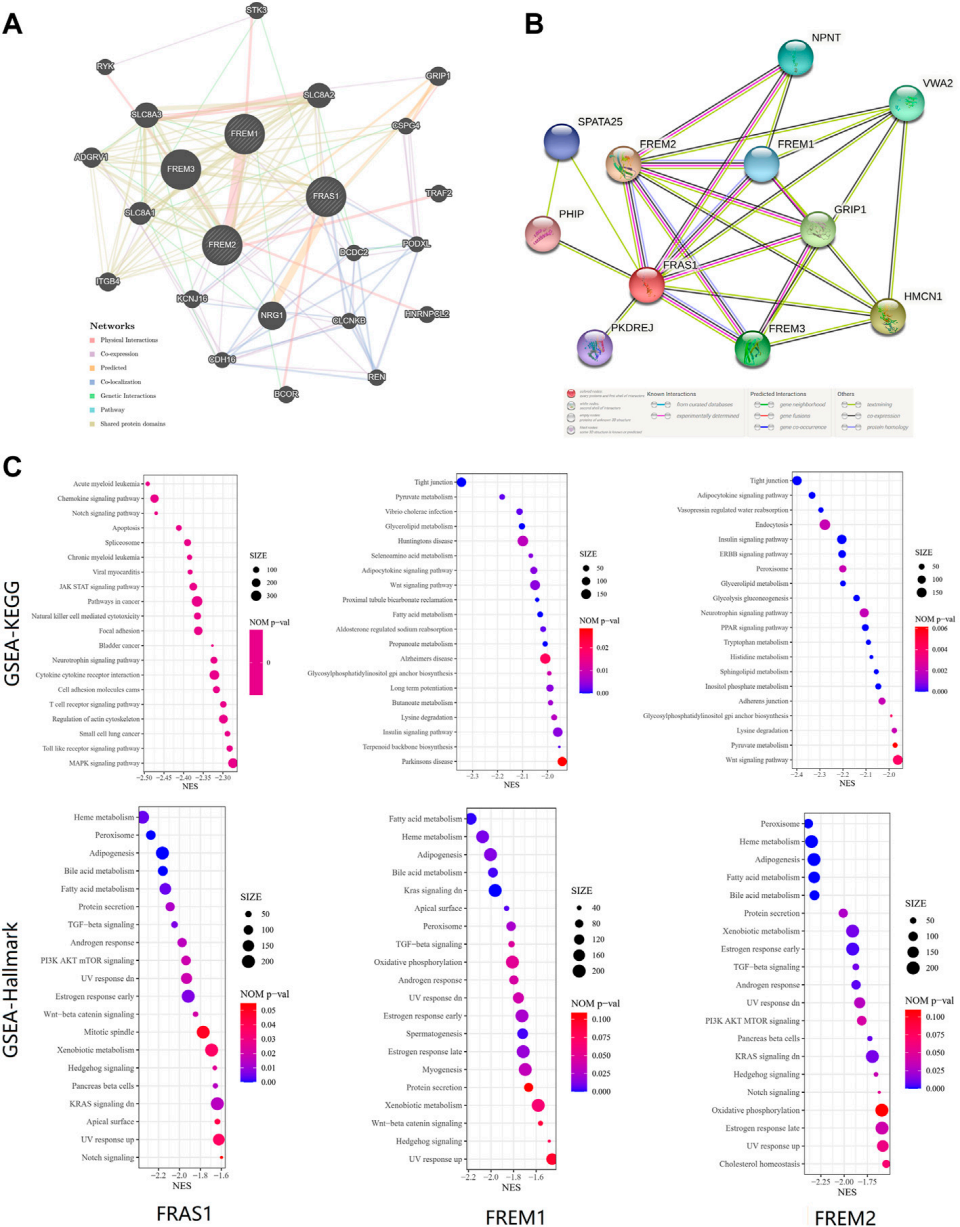
FRAS1/FREM-related gene enrichment, pathway analysis and function profiles. **(A)**PPI network analysis of FRAS1/FREM family members and their 20 co-regulated hub genes conducted by GeneMANIA. **(B)** STRING protein network map of experimentally determined FRAS1/FREM-binding proteins. **(C)** Significant gene set enrichment analysis (GSEA) results of FRAS1, FREM1 and FREM2 including KEGG pathways and Hallmark pathways.

Finally, to further investigate the molecular mechanism of the FRAS1, FREM1 and FREM2 in tumorigenesis, we filtered out the known FRAS1/FREM-interacting proteins and the FRAS1/FREM expression correlated genes for a series of pathway enrichment analyses. PPI network analysis of FRAS1/FREM family members and their 20 related genes was conducted by GeneMANIA (Figure 10A). In addition, using the STRING tool, we acquired a total of eight, experimentally detected FRAS1/FREM-binding proteins. Figure 10B shows the interaction network of these 11 proteins.

To investigate the biological significance of FRAS1, FREM1 and FREM2 expression in KIRC, we conducted GESA. The top 20 items of GO functional annotation (BP: biological process, MF: molecular function), the Kyoto Encyclopedia of Genes and Genomes (KEGG) and Hallmark were shown in Supplementary Figure S7; Figure 10C. The results of BP revealed that FRAS1, FREM1 and FREM2 were mainly enriched in regulation of mRNA, protein stabilization, transport, catabolic and methylation (Supplementary Figure S7A). The results of MF revealed that FRAS1, FREM1 and FREM2 were mainly enriched in regulation of expression, transport activity and specific epigenetic modification enzymatic activities (Supplementary Figure S7B). The results of KEGG and Hallmark revealed that FRAS1, FREM1 and FREM2 were mainly enriched in several signalling pathways (TGF-beta signaling, PI3K AKT mTOR signaling, Wnt-beta catenin signaling, Kras signaling, Hedgehog signaling and Notch signaling) and metabolic pathways (Heme metabolism, Fatty acid metabolism, Bile acid metabolism, Xenobiotic metabolism) (Figure 10C).

### Relationship between FRAS1/FREM expression and drug sensitivity

**FIGURE 11:**
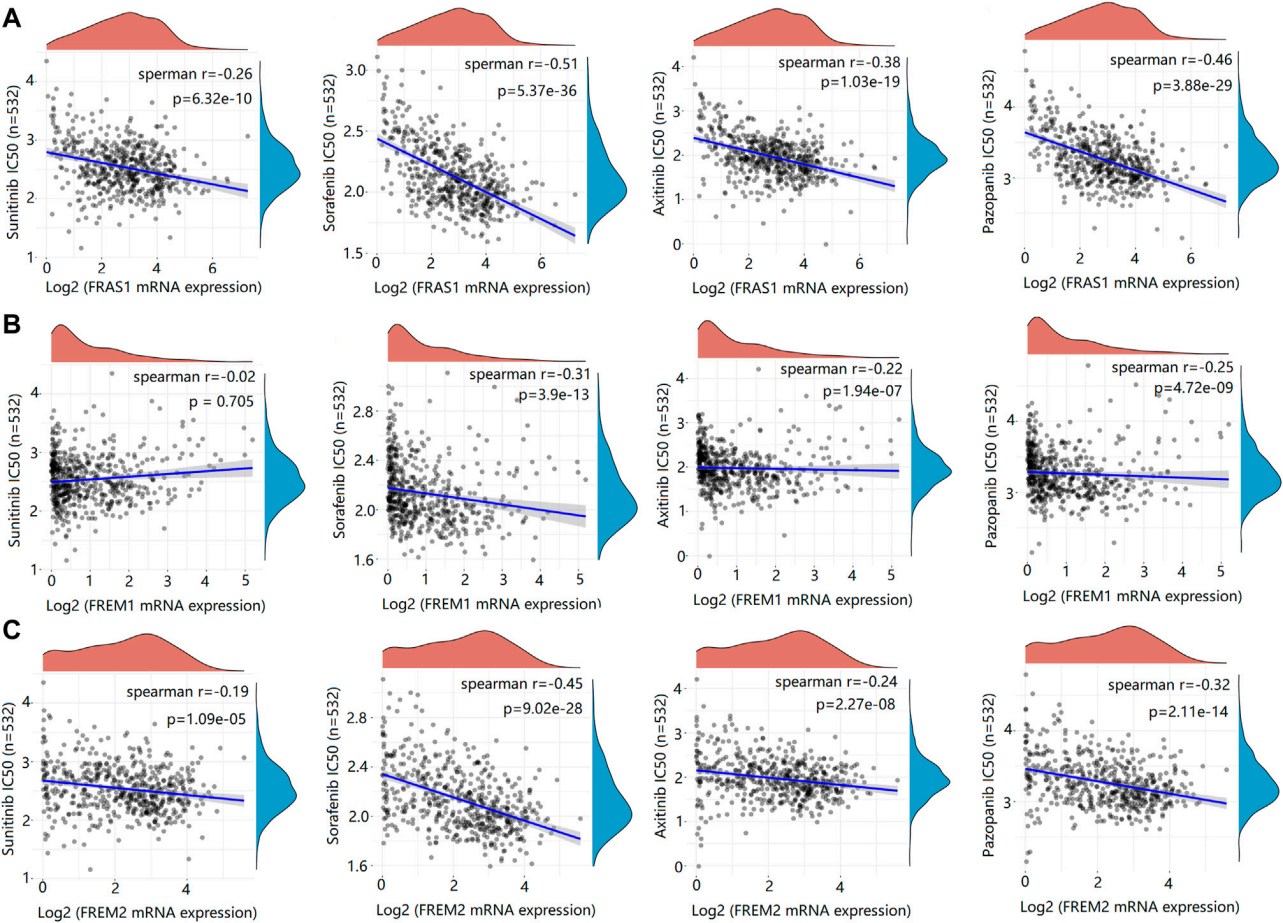
Drug sensitivity analysis of IC50 score and FRAS1/FREM expression. **(A)** Spearman correlation analysis of IC50 score and FRAS1 expression including four common targeted therapeutic drugs (sunitinib, sorafenib, axitinib and pazopanib) in KIRC. **(B)** Spearman correlation analysis of IC50 score and FREM1 expression. **(C)** Spearman correlation analysis of IC50 score and FREM2 expression.

Genetic alterations affect the drug sensitivity of cancer to clinical treatment and therefore are potential biomarkers for drug screening (Kim and Cho, 2022; Li et al., 2022). Therefore, we question the association between mRNA expression levels of FRAS1, FREM1 and FREM2 and patient sensitivity to four common targeted therapeutic drugs (sunitinib, sorafenib, axitinib and pazopanib). Based on the GDSC database, we performed a correlation analysis between gene expression level and drug sensitivity of the above four drugs in KIRC. We found that the IC50s of sunitinib (*r* = −0.26, *p* = 6.32e-10), sorafenib (*r* = −0.51, *p* = 5.37e-36), axitinib (*r* = −0.38, *p* = 1.03e-19) and pazopanib (*r* = −0.46, *p* = 3.88e-29) were significantly negatively correlated with FRAS1 expression (Figures 10A–D). Similarly, the IC50s of sunitinib (r = −0.19, *p* = 1.09e-05), sorafenib (r = −0.45, *p* = 9.02e-28), axitinib (r = −0.24, *p* = 2.27e-08) and pazopanib (r = −0.32, *p* = 2.11e-14) were also significantly negatively correlated with FRAS1 expression (Figures 11A–D). These results indicated that the expression level of FRAS1 and FREM2 interact with the sensitivity of targeted therapeutic drugs.

## Discussion

As one of the major components of TME, the ECM plays multiple crucial roles during tumorigenesis. The dysregulation of ECM is a remarkable feature of cancer. Growing studies have shown that ECM-related proteins may modulate the migration and invasion of cancer cells through related signaling pathways (Huang et al., 2021; Romani et al., 2021). More importantly, the deposition, reconstruction, and cross-linking of ECM can reprogram the local microenvironment and regulate the pro- and antitumor immune responses upon the stimulation of different ECM-related proteins, leading to aberrant mechanotransduction and further malignant transformation (Koliaraki et al., 2020; Nazemi and Rainero, 2020; Huang et al., 2021). As a special type of ECM, BM also presents the major barrier cancer cells have to overcome multiple times to form metastases (Stuelten et al., 2018; Reuten et al., 2021). Therefore, a comprehensive understanding of the dysregulation of the BM in the TME would contribute to the discovery of promising therapeutic targets for cancer treatment.

FRAS1, comprising 4,010 amino acids, is encoded by the FRAS1 gene located at the chromosome 4q21.21. FREM1 comprises 2,197 residues and resides on chromosome 9p22.3. In between, FREM2 consists of 3,160 amino acids and is encoded by the FREM2 gene located at the chromosome 13q13.3. FRAS1 and FREM2 are produced by epithelial cells as membrane proteins, while FREM1 is produced by mesenchymal cells as a secreted protein (Kiyozumi et al., 2012). These three proteins meet together at BM and form an independent ternary complex, supposedly having a similar function to collagen VII. FRAS1/FREM share common polypeptide repetitive motifs with possible interactive and organizing functions, and contribute to embryonic epithelial–mesenchymal integrity (Pavlakis et al., 2011; Kiyozumi et al., 2012).

FRAS1/FREM family are involved in the progression of several cancers. Zhan et al. (2014) reported that FRAS1 knockdown reduced non-small cell lung cancer A549 cells migration and invasion ability and Umeda et al. (2020) reported that knockout of FRAS1 inhibited liver metastasis of gastric cancer. The analysis of a xenograft model of human endometrial cancer found that FRAS1 might also serve as a potential diagnostic marker (Xu et al., 2012). FREM1 was reported to be associated with the favorable prognosis of breast carcinoma patients and negatively correlated with tumor stages in KIRC, while FREM2 was reported to be associated with favorable prognosis of patients with isocitrate dehydrogenase (IDH)- wild-type glioblastoma (Jovcevska et al., 2019; Li H. N. et al., 2020; Luo et al., 2020). Additionally, the polymorphism of FRAS1 was involved in various malignancies. For example, miR-1 targeting FRAS1 was downregulated in sunitinib resistance renal cancer cell (Butz et al., 2018). The rs1910301, the promoter region of FRAS1, was found to be a candidate SNP associated with lethal prostate cancer (Wang et al., 2021). The rs150303591, the nearby genomic loci of FRAS1 was also involved in drug resistance of ovarian cancer to carboplatin (Fridley et al., 2016). Yet, none of them in KIRC has been systematically studied.

Our study revealed that the mRNA and protein expression levels of FRAS1/FREM1 were significantly downregulated in KIRC tissues than in normal tissues. Besides, FRAS1, FREM1 and FREM2 mRNA and protein expressions were correlated with the clinicopathological characteristics (pathological stage, grade and tumor metastasis status) of the patients with KIRC. The expression levels of FRAS1 and FREM2 were low in the matestasis of KIRC tissues and might be associated with the number of pulmonary matastases. Interestingly, FRAS1 and FREM2 expression decreased in the stage I and stage II patients who had a high *propensity* to metastasise, which means FRAS1 and FREM2 could be tumor metastasis markers of KIRC. In addition, we report that FRAS1/FREM expression correlated with the prognosis of KIRC. Elevated FRAS1, FREM1 and FREM2 expression levels were found to be significantly related to a better OS, DSS and PFS in KIRC. Multivariate Cox proportional-hazards regression adjusted for clinical parameters (age, gender, race, TNM stage and grade) still suggested that FRAS1 and FREM2 could be independent risk factors for better prognosis. Thus, these results above indicated that FRAS1 and FREM2 might be used as potential biomarkers of diagnosis and prognosis in KIRC.

TMB is a potential pan-cancer predictive biomarker of immune checkpoint inhibitor response in most cancers (Jardim et al., 2021). MSI and MMR deficiency could also serve as an potential biomarker and predict the efficacy of immune-checkpoint inhibitors (ICI) (Baretti and Le, 2018). Our results show that FRAS1/FREM expression were positively correlated with essential MMR signatures in KIRC. In addation, FRAS1 and FREM2 expression were positively correlated with high MSI in KIRC, while FREM1 expression was negatively correlated with low TMB in KIRC. However, the correlation coefficients between FRAS1/FREM and TMB, as well as MSI, were below 0.5, suggesting that they were not sufficient to independently predict the patient’s response to immune checkpoint blockade efficacy.

Tumor immune infiltrating cells migrate from blood to tumor tissues and can antagonize or promote tumor occurrence and development. Cancer immunotherapy activates the immune system to specifically target malignant cells (Garner and de Visser, 2020). Previous research has often focused on CD8^+^ cytotoxic T cells, however, CD4^+^ T cells have gained attention in the field, as they are not only essential to promote help to CD8^+^ T cells, but are also able to kill tumor cells. Therefore, immunotherapy approaches have shifted from only stimulating CD8^+^ T cells to targeting and assessing CD4^+^ subsets and increasing numbers of clinical studies have demonstrated that targeting CD4^+^ T cells is safe and effective (Li T. et al., 2020; Cachot et al., 2021; Oh and Fong, 2021). Our study revealed that the FRAS1/FREM genes expression levels in KIRC were positively correlated with the infiltration level of CD4^+^ T cells and negatively correlated with the infiltration level of CD4^+^ T cell subsets (such as Th1, Tfh and Tregs). CD4^+^ T cells are now recognized as essential and pleiotropic effectors in the antitumor immune response, while various CD4^+^ T cell subsets play an antagonistic role in the antitumor immune response. Thus FRAS1/FREM might play an important part in recruitment and regulation of immune infiltrating cells, especially CD4^+^ T cells and its corresponding subsets (Th1, Th2, Tfh and Tregs) and macrophages. It will be interesting to investigate whether FRAS1/FREM might serve as new targets for the development of various cancer immunotherapies.

Another main finding of our study is that multiple factors such as genetic changes, epigenetic regulation, transcriptional regulation and translation regulation can synergistically be the potential mechanisms through which FRAS1/FREM exerts antitumor effects. As an a central epigenetic modification of the human genome, the changes of DNA methylation in cancer have been heralded as promising targets for the development of powerful diagnostic, prognostic, and predictive biomarkers (Dor and Cedar, 2018; Koch et al., 2018; Pan et al., 2021). Our study explored the relationship between FRAS1, FREM1 and FREM2 promoter methylation and cancer for the first time. We found that FRAS1 and FREM2 gene expression levels were significantly correlated with DNA promoter methylation and three DNA methyltransferases (DNMT1, DNMT2 and DNMT3A). This may partially explain the differential mRNA expression of FRAS1/FREM between tumor tissues and normal tissues, while the somatic mutation rates of them in KIRC were low. More importantly, the correlations of FRAS1 and FREM2 single CpG methylation levels with clinical prognosis revealed that they could serve as biomarkers of prognosis in patients with KIRC. Consistent with the conclusion stated above, the results of BP and MF by GSEA also revealed that FRAS1, FREM1 and FREM2 were mainly enriched in the process of transcriptional regulation, post-transcriptional regulation, and regulation of specific epigenetic modification enzymatic activities.

The oncogenic developmental signalling pathways such as the Notch, WNT, Hedgehog and Hippo are crucial for the development Cancer stem cells (CSC), which have important roles in tumour development, relapse and metastasis. As such, therapeutics targeting the above pathways are prime targets for anti-CSC therapy—with some success in certain tumors (Saygin et al., 2019; Clara et al., 2020). The crosstalk between the above pathways and other tumorigenic pathways (e.g., NF-κB, KRAS–RAF–MAPK and PI3K–AKT–mTOR) have also hinted at their profoundly complex roles in cancer (Pelullo et al., 2019; Clara et al., 2020). Moreover, the tumor microenvironment and the ECM can regulate cell metabolism, such as glucose metabolism and lipid synthesis (Nazemi and Rainero, 2020; Romani et al., 2021). Indeed, aberrant activation of PI3K/AKT and Ras signaling pathways can facilitate constant glucose uptake, mTOR can also induce anabolic processes such as protein, nucleotide, and lipid biosynthesis (Nazemi and Rainero, 2020). Our results of KEGG and Hallmark by GSEA revealed that FRAS1, FREM1 and FREM2 can potentially impact cancer etiology or pathogenesis by functioning in oncogenic signalling pathways (TGF-β signaling, PI3K AKT mTOR signaling, Wnt-β catenin signaling, Kras signaling, Hedgehog signaling and Notch signaling) and metabolic pathways (Heme metabolism, Fatty acid metabolism, Bile acid metabolism, Xenobiotic metabolism). It will be very interesting to find the potential targets that are involved in these signaling and potentially interact with FRAS1/FREM in future studies. A better understanding of the interplay between FRAS1/FREM and the tumor microenvironment might be the key to unlock a new era of oncological treatments and proposes new therapeutic targets for KIRC.

Through drug sensitivity analysis, we found that high FRAS1 and FREM2 expression were negatively correlated with IC50 values of four common targeted therapeutic drugs (sunitinib, sorafenib, axitinib and pazopanib) which indicated that the measurement of FRAS1 and FREM2 expression level can be a reliable predictor of targeted therapeutic drug response, highlighting the potential as an anticancer drug target. These results will help us better understand how the ECM protein in TME can benefit cancer treatment, and guide the drug selection in patients with multiline treatment resistance.

However, there are limitations to our study. First, while bioinformatics has the advantages of large sample size, simplicity and low cost, the biases caused by the confounders might exist. Second, our analyses are mainly based on TCGA database and some results are further validated by GEO database, CPTAC database and RT-PCR results. Even so, further experiments *in vivo* and *in vitro* should be still needed. Third, although a serious of function annotations and enrichment analysis were investigated in our study, the detailed molecular biological mechanisms of FRAS1/FREM in KIRC need to be further validated.

In summary, our first pan-cancer analyses indicated that the FRAS1/FREM genes and proteins were differentially expressed between tumor and normal tissues. Moreover, elevated FRAS1/FREM expression levels were significantly correlated with cancer progression (pathological stage, pathological grade and tumor metastasis status), poor survival (OS, DSS and PFS), immune infiltrations (MMR, TMB, MSI and CD4^+^ T cells subsets) and DNA methylation in KIRC patients, sharing the potential as efficient markers of the prognostic value of KIRC and potential targets in the development of anti-KIRC therapeutics.

## Data Availability

The original contributions presented in the study are included in the article/Supplementary Material, further inquiries can be directed to the corresponding author.
