# Supplementary material for: Comprehensive analysis of FRAS1/FREM family as potential biomarkers and therapeutic targets in renal clear cell carcinoma
Source: Front Pharmacol. 2022 Sep 29;13:972934. doi: 10.3389/fphar.2022.972934 (PMC9558830; doi:10.3389/fphar.2022.972934)
Supplement: Supplementary file 1 [file DataSheet1.PDF]

Figure Legend

FIGURE S1 | Pan-cancer expression analysis of FREM1 in human tumors vs normal tissues. (A) Differential expression of FREM1 in the Oncomine database. (B) The expression profile of FREM1 in pan-cancer analysis by TIMER2.0. (C) The expression profile of FREM1 in pan-cancer analysis from TCGA database and GTEx database. (*P < 0.05, **P < 0.01, ***P < 0.001).

FIGURE S2 | Pan-cancer expression analysis of FREM2 in human tumors vs normal tissues. (A) Differential expression of FREM2 in the Oncomine database. (B) The expression profile of FREM2 in pan-cancer analysis by TIMER2.0. (C) The expression profile of FREM2 in pan-cancer analysis from TCGA database and GTEx database. (*P < 0.05, **P < 0.01, ***P < 0.001)

FIGURE S3 | The correlations between FRAS1/FREM and essential genes involved in MMR (MLH1, MSH2, MSH6, PMS2 and EpCAM) in multiple cancers (*P < 0.05, **P < 0.01, ***P < 0.001).

FIGURE S4 | The correlations between FRAS1/FREM and TMB, MSI in multiple cancers (*P < 0.05, **P < 0.01, ***P < 0.001).

FIGURE S5 | Relationship between FRAS1/FREM expression and immune cell infiltration in KIRC.

FIGURE S6 | The prognostic value of DNA methylation of FRAS1 and FREM2 in KIRC.

FIGURE S7 | Significant gene set enrichment analysis (GSEA) results of FRAS1, FREM1 and FREM2 including biological processes (BP), molecular function (MF).

Figure S1.

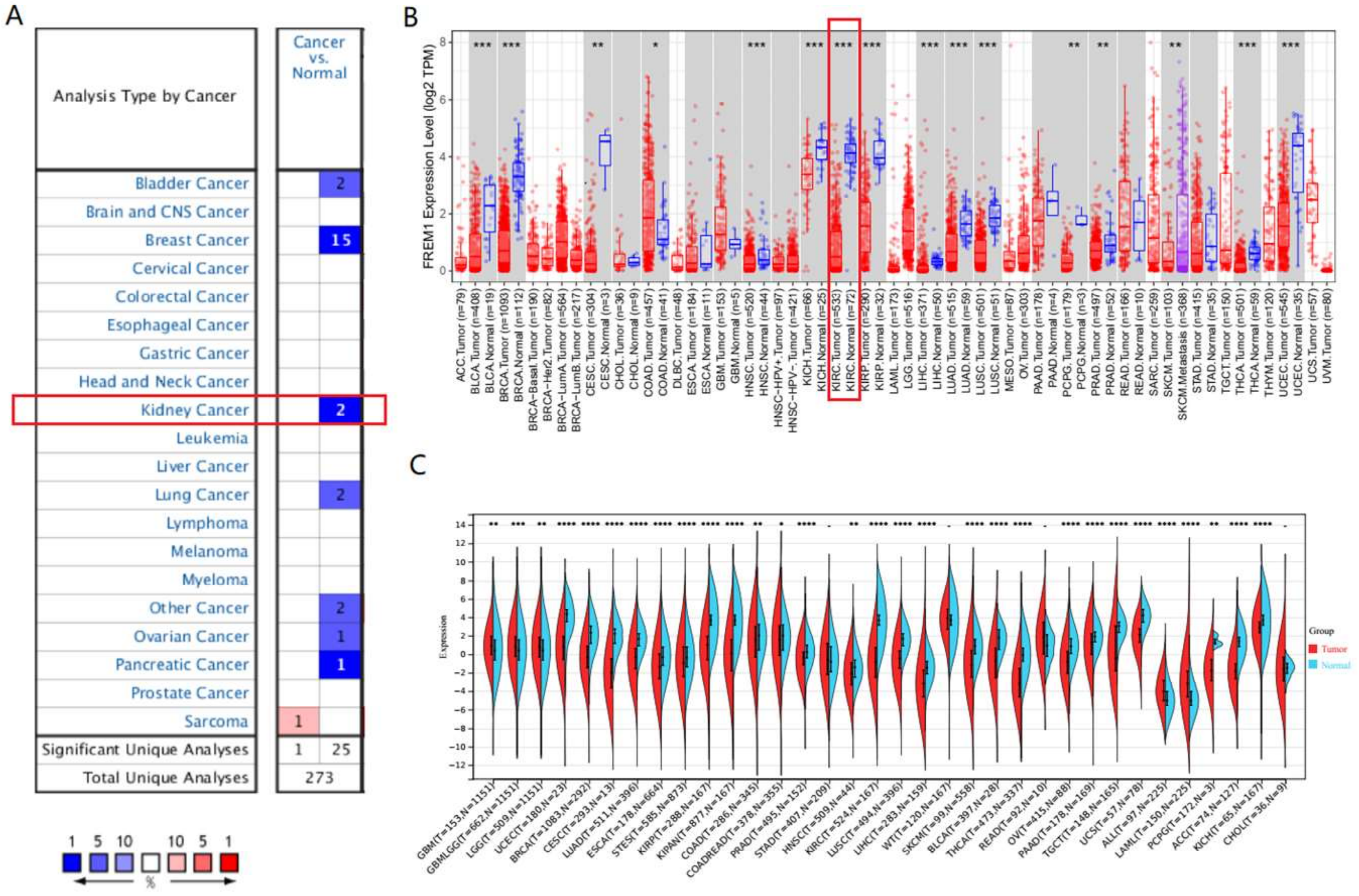
A
Analysis Type by Cancer
Cancer vs. Normal
Bladder Cancer 2
Brain and CNS Cancer
Breast Cancer 15
Cervical Cancer
Colorectal Cancer
Esophageal Cancer
Gastric Cancer
Head and Neck Cancer
Kidney Cancer 2
Leukemia
Liver Cancer
Lung Cancer 2
Lymphoma
Melanoma
Myeloma
Other Cancer 2
Ovarian Cancer 1
Pancreatic Cancer 1
Prostate Cancer
Sarcoma 1
Significant Unique Analyses 1 25
Total Unique Analyses 273
1 5 10 10 5 1
%
B
FREM1 Expression Level (log2 TPM)
C
Expression
Group
Tumor
Normal

Figure S2

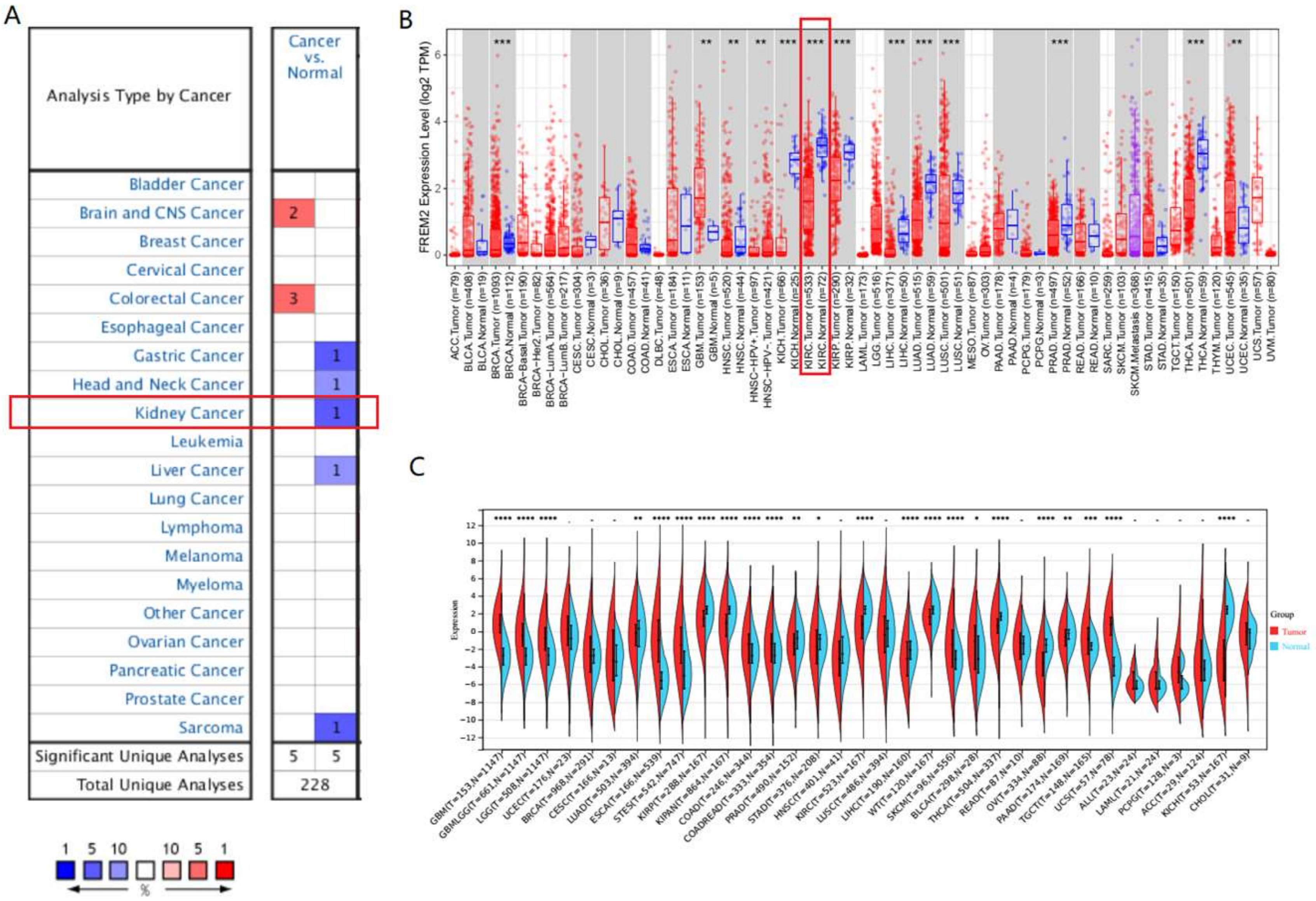
A
Analysis Type by Cancer
Cancer vs. Normal
Bladder Cancer
Brain and CNS Cancer
2
Breast Cancer
Cervical Cancer
Colorectal Cancer
3
Esophageal Cancer
Gastric Cancer
1
Head and Neck Cancer
1
Kidney Cancer
1
Leukemia
Liver Cancer
1
Lung Cancer
Lymphoma
Melanoma
Myeloma
Other Cancer
Ovarian Cancer
Pancreatic Cancer
Prostate Cancer
Sarcoma
1
Significant Unique Analyses
5
5
Total Unique Analyses
228
1 5 10 10 5 1
%
B
FREM2 Expression Level (log2 TPM)
C
Expression
Group
Tumor
Normal

Figure S3.

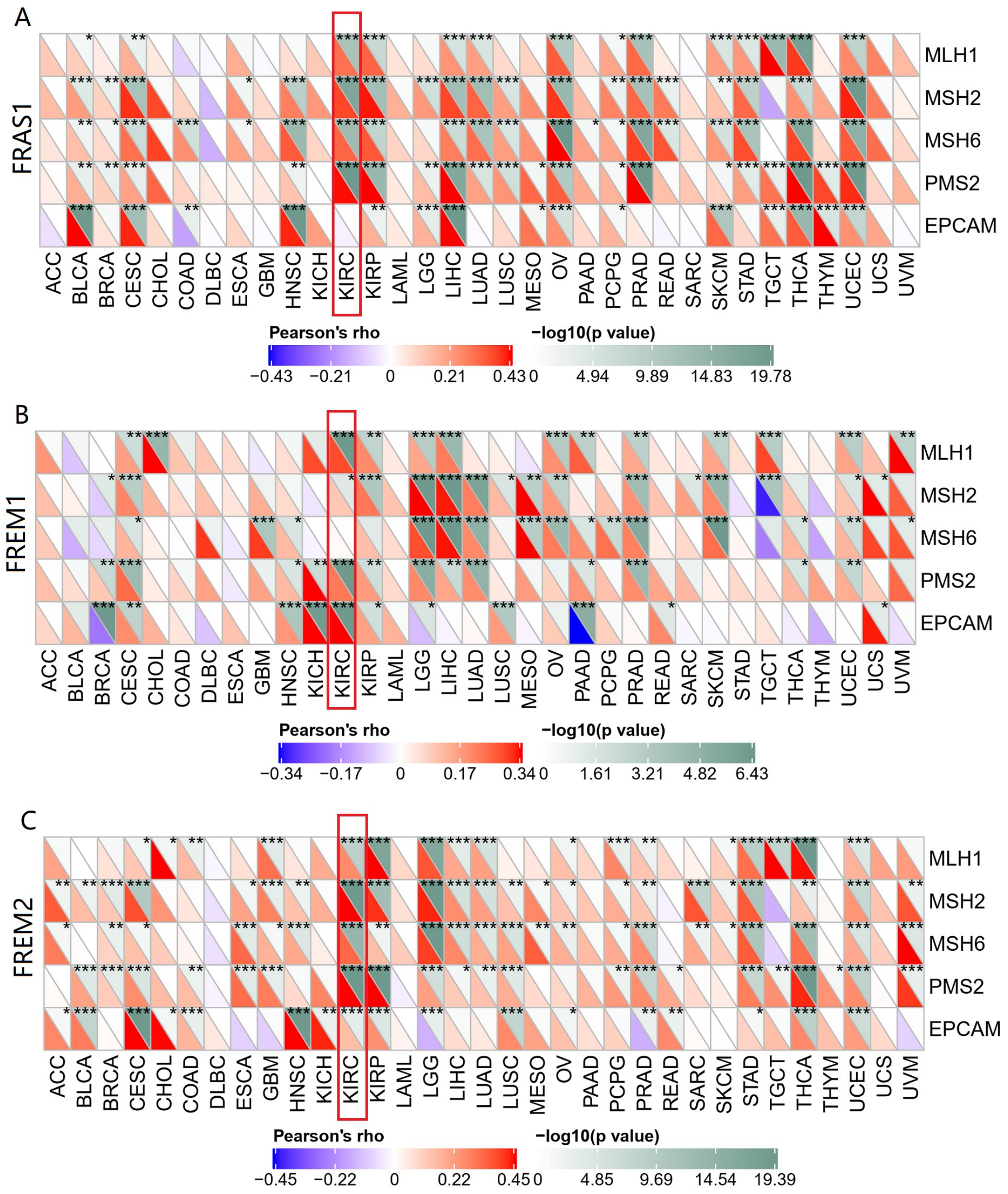
A
FRAS1
MLH1
MSH2
MSH6
PMS2
EPCAM
ACC BLCA BRCA CESC CHOL COAD DLBC ESCA GBM HNSC KICH KIRC KIRP LAML LGG LIHC LUAD LUSC MESO OV PAAD PCPG PRAD READ SARC SKCM STAD TGCT THCA THYM UCEC UCS UVM
Pearson's rho
−0.43 −0.21 0 0.21 0.43
−log10(p value)
0 4.94 9.89 14.83 19.78
B
FREM1
MLH1
MSH2
MSH6
PMS2
EPCAM
ACC BLCA BRCA CESC CHOL COAD DLBC ESCA GBM HNSC KICH KIRC KIRP LAML LGG LIHC LUAD LUSC MESO OV PAAD PCPG PRAD READ SARC SKCM STAD TGCT THCA THYM UCEC UCS UVM
Pearson's rho
−0.34 −0.17 0 0.17 0.34
−log10(p value)
0 1.61 3.21 4.82 6.43
C
FREM2
MLH1
MSH2
MSH6
PMS2
EPCAM
ACC BLCA BRCA CESC CHOL COAD DLBC ESCA GBM HNSC KICH KIRC KIRP LAML LGG LIHC LUAD LUSC MESO OV PAAD PCPG PRAD READ SARC SKCM STAD TGCT THCA THYM UCEC UCS UVM
Pearson's rho
−0.45 −0.22 0 0.22 0.45
−log10(p value)
0 4.85 9.69 14.54 19.39

Figure S4.

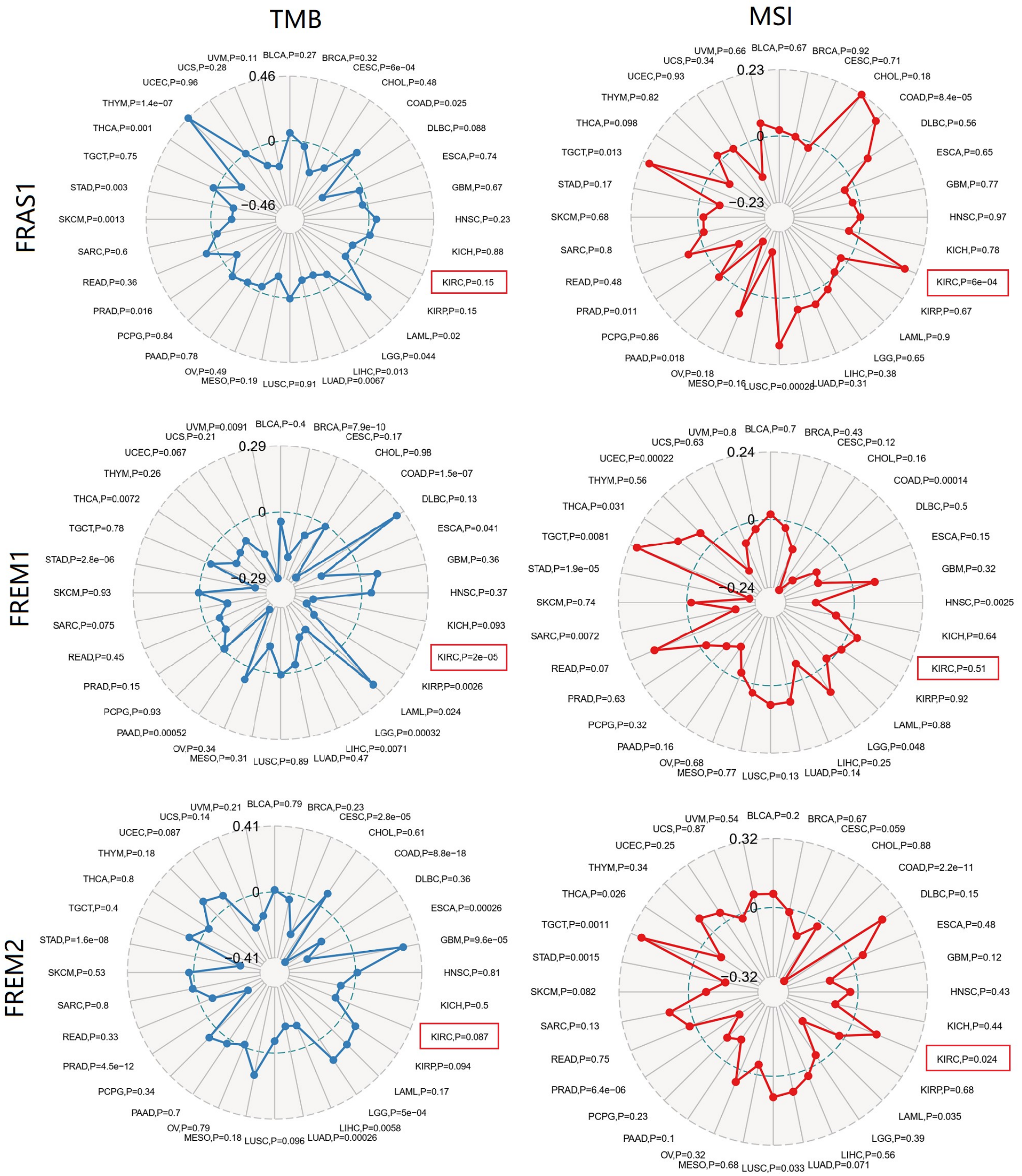
TMB
MSI
FRAS1
FREM1
FREM2
0.46
0
-0.46
BLCA,P=0.27
BRCA,P=0.32
CESC,P=6e-04
CHOL,P=0.48
COAD,P=0.025
DLBC,P=0.088
ESCA,P=0.74
GBM,P=0.67
HNSC,P=0.23
KICH,P=0.88
KIRC,P=0.15
KIRP,P=0.15
LAML,P=0.02
LGG,P=0.044
LIHC,P=0.013
LUAD,P=0.0067
LUSC,P=0.91
MESO,P=0.19
OV,P=0.49
PAAD,P=0.78
PCPG,P=0.84
PRAD,P=0.016
READ,P=0.36
SARC,P=0.6
SKCM,P=0.0013
STAD,P=0.003
TGCT,P=0.75
THCA,P=0.001
THYM,P=1.4e-07
UCEC,P=0.96
UCS,P=0.28
UVM,P=0.11
0.23
0
-0.23
BLCA,P=0.67
BRCA,P=0.92
CESC,P=0.71
CHOL,P=0.18
COAD,P=8.4e-05
DLBC,P=0.56
ESCA,P=0.65
GBM,P=0.77
HNSC,P=0.97
KICH,P=0.78
KIRC,P=6e-04
KIRP,P=0.67
LAML,P=0.9
LGG,P=0.65
LIHC,P=0.38
LUAD,P=0.31
LUSC,P=0.00028
MESO,P=0.16
OV,P=0.18
PAAD,P=0.018
PCPG,P=0.86
PRAD,P=0.011
READ,P=0.48
SARC,P=0.8
SKCM,P=0.68
STAD,P=0.17
TGCT,P=0.013
THCA,P=0.098
THYM,P=0.82
UCEC,P=0.93
UCS,P=0.34
UVM,P=0.66
0.29
0
-0.29
BLCA,P=0.4
BRCA,P=7.9e-10
CESC,P=0.17
CHOL,P=0.98
COAD,P=1.5e-07
DLBC,P=0.13
ESCA,P=0.041
GBM,P=0.36
HNSC,P=0.37
KICH,P=0.093
KIRC,P=2e-05
KIRP,P=0.0026
LAML,P=0.024
LGG,P=0.00032
LIHC,P=0.0071
LUAD,P=0.47
LUSC,P=0.89
MESO,P=0.31
OV,P=0.34
PAAD,P=0.00052
PCPG,P=0.93
PRAD,P=0.15
READ,P=0.45
SARC,P=0.075
SKCM,P=0.93
STAD,P=2.8e-06
TGCT,P=0.78
THCA,P=0.0072
THYM,P=0.26
UCEC,P=0.067
UCS,P=0.21
UVM,P=0.0091
0.24
0
-0.24
BLCA,P=0.7
BRCA,P=0.43
CESC,P=0.12
CHOL,P=0.16
COAD,P=0.00014
DLBC,P=0.5
ESCA,P=0.15
GBM,P=0.32
HNSC,P=0.0025
KICH,P=0.64
KIRC,P=0.51
KIRP,P=0.92
LAML,P=0.88
LGG,P=0.048
LIHC,P=0.25
LUAD,P=0.14
LUSC,P=0.13
MESO,P=0.77
OV,P=0.68
PAAD,P=0.16
PCPG,P=0.32
PRAD,P=0.63
READ,P=0.07
SARC,P=0.0072
SKCM,P=0.74
STAD,P=1.9e-05
TGCT,P=0.0081
THCA,P=0.031
THYM,P=0.56
UCEC,P=0.00022
UCS,P=0.63
UVM,P=0.8
0.41
0
-0.41
BLCA,P=0.79
BRCA,P=0.23
CESC,P=2.8e-05
CHOL,P=0.61
COAD,P=8.8e-18
DLBC,P=0.36
ESCA,P=0.00026
GBM,P=9.6e-05
HNSC,P=0.81
KICH,P=0.5
KIRC,P=0.087
KIRP,P=0.094
LAML,P=0.17
LGG,P=5e-04
LIHC,P=0.0058
LUAD,P=0.00026
LUSC,P=0.096
MESO,P=0.18
OV,P=0.79
PAAD,P=0.7
PCPG,P=0.34
PRAD,P=4.5e-12
READ,P=0.33
SARC,P=0.8
SKCM,P=0.53
STAD,P=1.6e-08
TGCT,P=0.4
THCA,P=0.8
THYM,P=0.18
UCEC,P=0.087
UCS,P=0.14
UVM,P=0.21
0.32
0
-0.32
BLCA,P=0.2
BRCA,P=0.67
CESC,P=0.059
CHOL,P=0.88
COAD,P=2.2e-11
DLBC,P=0.15
ESCA,P=0.48
GBM,P=0.12
HNSC,P=0.43
KICH,P=0.44
KIRC,P=0.024
KIRP,P=0.68
LAML,P=0.035
LGG,P=0.39
LIHC,P=0.56
LUAD,P=0.071
LUSC,P=0.033
MESO,P=0.68
OV,P=0.32
PAAD,P=0.1
PCPG,P=0.23
PRAD,P=6.4e-06
READ,P=0.75
SARC,P=0.13
SKCM,P=0.082
STAD,P=0.0015
TGCT,P=0.0011
THCA,P=0.026
THYM,P=0.34
UCEC,P=0.25
UCS,P=0.87
UVM,P=0.54

Figure S5.

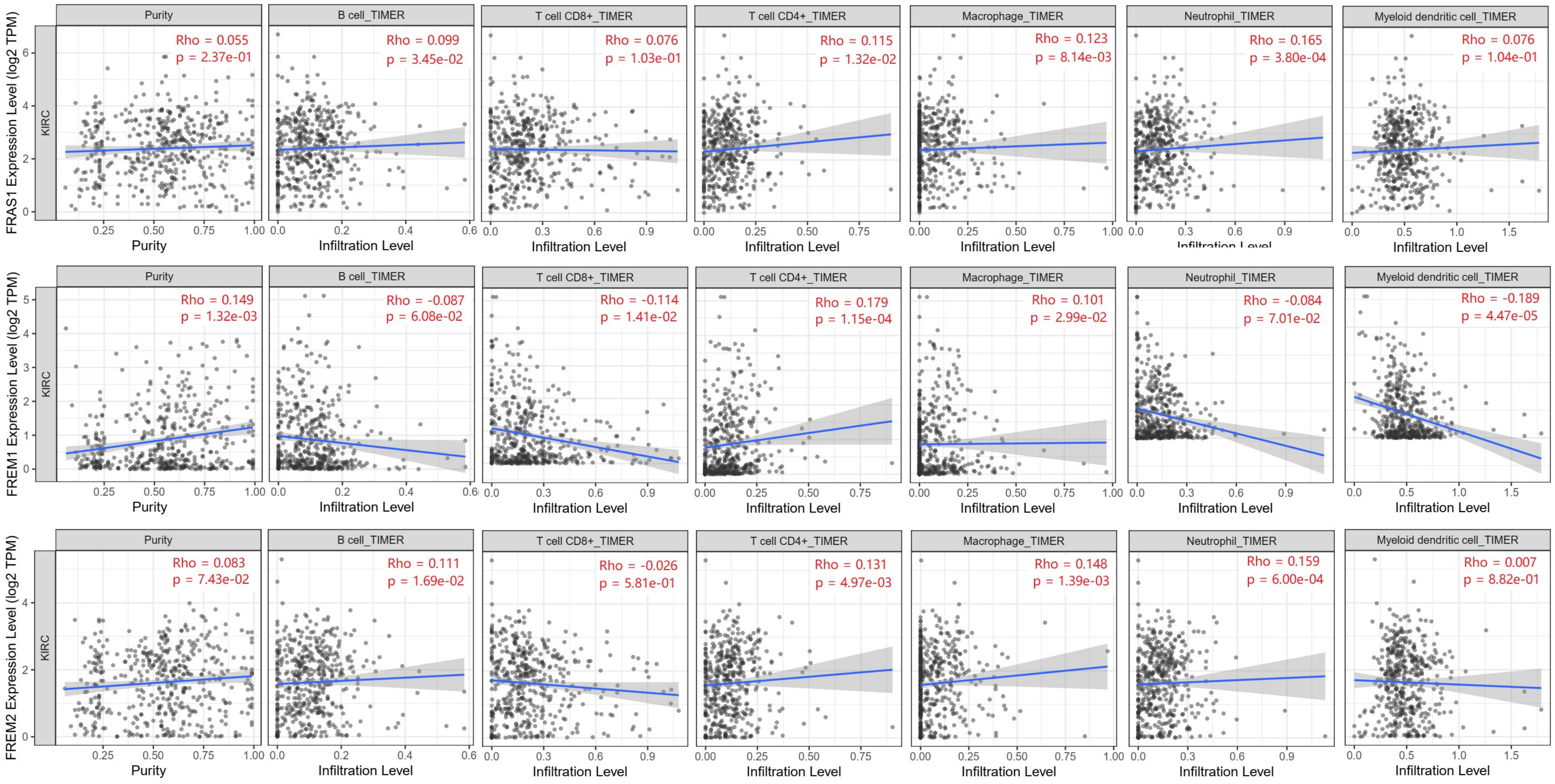
FRAS1 Expression Level (log2 TPM)
FREM1 Expression Level (log2 TPM)
FREM2 Expression Level (log2 TPM)
KIRC
Purity
B cell_TIMER
T cell CD8+_TIMER
T cell CD4+_TIMER
Macrophage_TIMER
Neutrophil_TIMER
Myeloid dendritic cell_TIMER
Rho = 0.055
p = 2.37e-01
Rho = 0.099
p = 3.45e-02
Rho = 0.076
p = 1.03e-01
Rho = 0.115
p = 1.32e-02
Rho = 0.123
p = 8.14e-03
Rho = 0.165
p = 3.80e-04
Rho = 0.076
p = 1.04e-01
Rho = 0.149
p = 1.32e-03
Rho = -0.087
p = 6.08e-02
Rho = -0.114
p = 1.41e-02
Rho = 0.179
p = 1.15e-04
Rho = 0.101
p = 2.99e-02
Rho = -0.084
p = 7.01e-02
Rho = -0.189
p = 4.47e-05
Rho = 0.083
p = 7.43e-02
Rho = 0.111
p = 1.69e-02
Rho = -0.026
p = 5.81e-01
Rho = 0.131
p = 4.97e-03
Rho = 0.148
p = 1.39e-03
Rho = 0.159
p = 6.00e-04
Rho = 0.007
p = 8.82e-01
Purity
Infiltration Level

Figure 6.

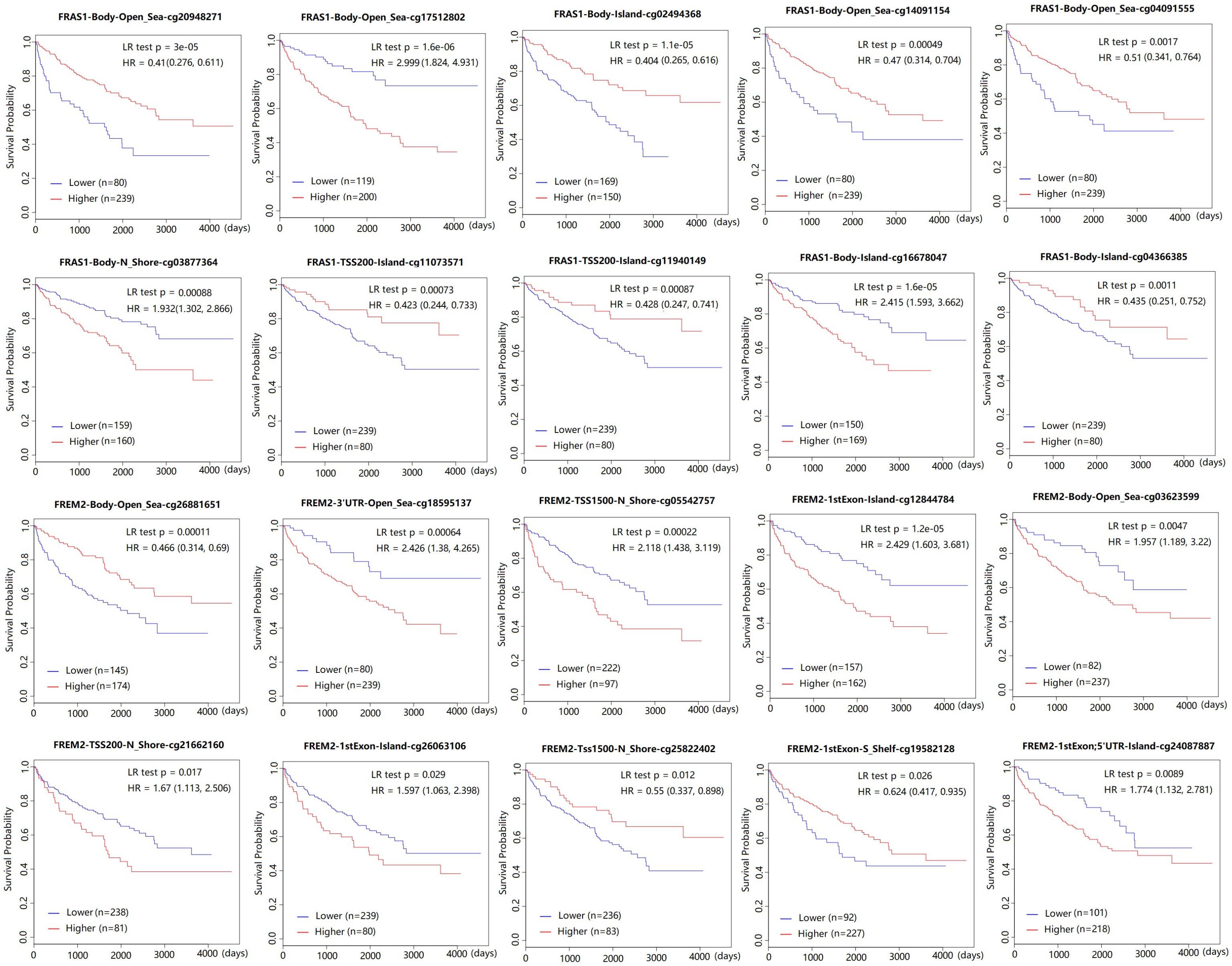
FRAS1-Body-Open_Sea-cg20948271
LR test p = 3e-05
HR = 0.41(0.276, 0.611)
Lower (n=80)
Higher (n=239)
FRAS1-Body-Open_Sea-cg17512802
LR test p = 1.6e-06
HR = 2.999 (1.824, 4.931)
Lower (n=119)
Higher (n=200)
FRAS1-Body-Island-cg02494368
LR test p = 1.1e-05
HR = 0.404 (0.265, 0.616)
Lower (n=169)
Higher (n=150)
FRAS1-Body-Open_Sea-cg14091154
LR test p = 0.00049
HR = 0.47 (0.314, 0.704)
Lower (n=80)
Higher (n=239)
FRAS1-Body-Open_Sea-cg04091555
LR test p = 0.0017
HR = 0.51 (0.341, 0.764)
Lower (n=80)
Higher (n=239)
FRAS1-Body-N_Shore-cg03877364
LR test p = 0.00088
HR = 1.932(1.302, 2.866)
Lower (n=159)
Higher (n=160)
FRAS1-TSS200-Island-cg11073571
LR test p = 0.00073
HR = 0.423 (0.244, 0.733)
Lower (n=239)
Higher (n=80)
FRAS1-TSS200-Island-cg11940149
LR test p = 0.00087
HR = 0.428 (0.247, 0.741)
Lower (n=239)
Higher (n=80)
FRAS1-Body-Island-cg16678047
LR test p = 1.6e-05
HR = 2.415 (1.593, 3.662)
Lower (n=150)
Higher (n=169)
FRAS1-Body-Island-cg04366385
LR test p = 0.0011
HR = 0.435 (0.251, 0.752)
Lower (n=239)
Higher (n=80)
FREM2-Body-Open_Sea-cg26881651
LR test p = 0.00011
HR = 0.466 (0.314, 0.69)
Lower (n=145)
Higher (n=174)
FREM2-3'UTR-Open_Sea-cg18595137
LR test p = 0.00064
HR = 2.426 (1.38, 4.265)
Lower (n=80)
Higher (n=239)
FREM2-TSS1500-N_Shore-cg05542757
LR test p = 0.00022
HR = 2.118 (1.438, 3.119)
Lower (n=222)
Higher (n=97)
FREM2-1stExon-Island-cg12844784
LR test p = 1.2e-05
HR = 2.429 (1.603, 3.681)
Lower (n=157)
Higher (n=162)
FREM2-Body-Open_Sea-cg03623599
LR test p = 0.0047
HR = 1.957 (1.189, 3.22)
Lower (n=82)
Higher (n=237)
FREM2-TSS200-N_Shore-cg21662160
LR test p = 0.017
HR = 1.67 (1.113, 2.506)
Lower (n=238)
Higher (n=81)
FREM2-1stExon-Island-cg26063106
LR test p = 0.029
HR = 1.597 (1.063, 2.398)
Lower (n=239)
Higher (n=80)
FREM2-Tss1500-N_Shore-cg25822402
LR test p = 0.012
HR = 0.55 (0.337, 0.898)
Lower (n=236)
Higher (n=83)
FREM2-1stExon-S_Shelf-cg19582128
LR test p = 0.026
HR = 0.624 (0.417, 0.935)
Lower (n=92)
Higher (n=227)
FREM2-1stExon;5'UTR-Island-cg24087887
LR test p = 0.0089
HR = 1.774 (1.132, 2.781)
Lower (n=101)
Higher (n=218)
Survival Probability
0.0
0.2
0.4
0.6
0.8
1.0
0
1000
2000
3000
4000
(days)

Figure S7.

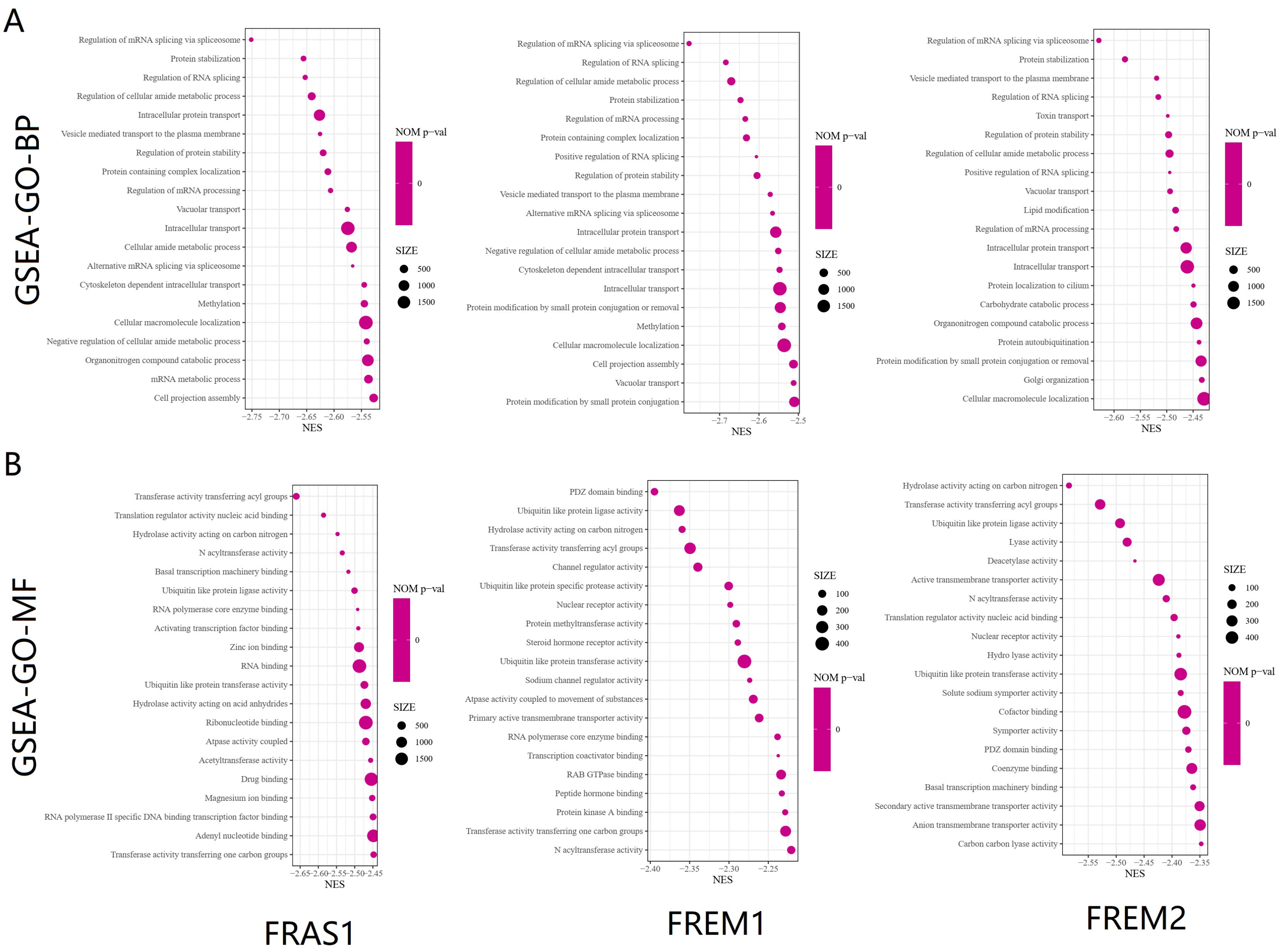
A
GSEA-GO-BP
Regulation of mRNA splicing via spliceosome
Protein stabilization
Regulation of RNA splicing
Regulation of cellular amide metabolic process
Intracellular protein transport
Vesicle mediated transport to the plasma membrane
Regulation of protein stability
Protein containing complex localization
Regulation of mRNA processing
Vacuolar transport
Intracellular transport
Cellular amide metabolic process
Alternative mRNA splicing via spliceosome
Cytoskeleton dependent intracellular transport
Methylation
Cellular macromolecule localization
Negative regulation of cellular amide metabolic process
Organonitrogen compound catabolic process
mRNA metabolic process
Cell projection assembly
−2.75 −2.70 −2.65 −2.60 −2.55
NES
NOM p−val
0
SIZE
500
1000
1500
Regulation of mRNA splicing via spliceosome
Regulation of RNA splicing
Regulation of cellular amide metabolic process
Protein stabilization
Regulation of mRNA processing
Protein containing complex localization
Positive regulation of RNA splicing
Regulation of protein stability
Vesicle mediated transport to the plasma membrane
Alternative mRNA splicing via spliceosome
Intracellular protein transport
Negative regulation of cellular amide metabolic process
Cytoskeleton dependent intracellular transport
Intracellular transport
Protein modification by small protein conjugation or removal
Methylation
Cellular macromolecule localization
Cell projection assembly
Vacuolar transport
Protein modification by small protein conjugation
−2.7 −2.6 −2.5
NES
NOM p−val
0
SIZE
500
1000
1500
Regulation of mRNA splicing via spliceosome
Protein stabilization
Vesicle mediated transport to the plasma membrane
Regulation of RNA splicing
Toxin transport
Regulation of protein stability
Regulation of cellular amide metabolic process
Positive regulation of RNA splicing
Vacuolar transport
Lipid modification
Regulation of mRNA processing
Intracellular protein transport
Intracellular transport
Protein localization to cilium
Carbohydrate catabolic process
Organonitrogen compound catabolic process
Protein autoubiquitination
Protein modification by small protein conjugation or removal
Golgi organization
Cellular macromolecule localization
−2.60 −2.55 −2.50 −2.45
NES
NOM p−val
0
SIZE
500
1000
1500
B
GSEA-GO-MF
Transferase activity transferring acyl groups
Translation regulator activity nucleic acid binding
Hydrolase activity acting on carbon nitrogen
N acyltransferase activity
Basal transcription machinery binding
Ubiquitin like protein ligase activity
RNA polymerase core enzyme binding
Activating transcription factor binding
Zinc ion binding
RNA binding
Ubiquitin like protein transferase activity
Hydrolase activity acting on acid anhydrides
Ribonucleotide binding
Atpase activity coupled
Acetyltransferase activity
Drug binding
Magnesium ion binding
RNA polymerase II specific DNA binding transcription factor binding
Adenyl nucleotide binding
Transferase activity transferring one carbon groups
−2.65 −2.60 −2.55 −2.50 −2.45
NES
NOM p−val
0
SIZE
500
1000
1500
PDZ domain binding
Ubiquitin like protein ligase activity
Hydrolase activity acting on carbon nitrogen
Transferase activity transferring acyl groups
Channel regulator activity
Ubiquitin like protein specific protease activity
Nuclear receptor activity
Protein methyltransferase activity
Steroid hormone receptor activity
Ubiquitin like protein transferase activity
Sodium channel regulator activity
Atpase activity coupled to movement of substances
Primary active transmembrane transporter activity
RNA polymerase core enzyme binding
Transcription coactivator binding
RAB GTPase binding
Peptide hormone binding
Protein kinase A binding
Transferase activity transferring one carbon groups
N acyltransferase activity
−2.40 −2.35 −2.30 −2.25
NES
SIZE
100
200
300
400
NOM p−val
0
Hydrolase activity acting on carbon nitrogen
Transferase activity transferring acyl groups
Ubiquitin like protein ligase activity
Lyase activity
Deacetylase activity
Active transmembrane transporter activity
N acyltransferase activity
Translation regulator activity nucleic acid binding
Nuclear receptor activity
Hydro lyase activity
Ubiquitin like protein transferase activity
Solute sodium symporter activity
Cofactor binding
Symporter activity
PDZ domain binding
Coenzyme binding
Basal transcription machinery binding
Secondary active transmembrane transporter activity
Anion transmembrane transporter activity
Carbon carbon lyase activity
−2.55 −2.50 −2.45 −2.40 −2.35
NES
SIZE
100
200
300
400
NOM p−val
0
FRAS1
FREM1
FREM2
